# Synthesis and evaluation of novel naphthol diazenyl scaffold based Schiff bases as potential antimicrobial and cytotoxic agents against human colorectal carcinoma cell line (HT-29)

**DOI:** 10.1186/s13065-019-0558-y

**Published:** 2019-04-02

**Authors:** Harmeet Kaur, Jasbir Singh, Balasubramanian Narasimhan

**Affiliations:** 1 Faculty of Pharmaceutical Sciences, Maharshi Dayanand University, Rohtak, 124001 India; 2 College of Pharmacy, Postgraduate Institute of Medical Sciences, Rohtak, 124001 India

**Keywords:** Schiff base, Antimicrobial, Diazenyl, Apoptosis, Cell cycle

## Abstract

**Background:**

In search of new antimicrobial and cytotoxic agents, a series of new naphthol diazenyl scaffold based Schiff bases (**NS1**–**NS23**) was efficiently synthesized by condensation of 2-hydroxy naphthaldehyde azo dyes with various substituted aromatic/heteroaromatic/aliphatic amines.

**Methodology:**

The synthesized derivatives were characterized by various physicochemical and spectral techniques and assessed for in vitro antimicrobial and cytotoxic potential against human colorectal carcinoma cell line (HT-29). The active derivatives were further evaluated for their apoptotic potential by Annexin-V/propidium iodide double staining assay using flow cytometer and analyzed for cell-cycle arrest studies.

**Results and conclusion:**

The derivative **NS-2** was found maximum active against *E*. *coli*, *S. enterica* and *B. subtilis*. The derivatives **NS-12**, **NS-15**, **NS-21**, and **NS-23** showed maximum antifungal activity against *A. fumigatus*. The maximum cytotoxicity was observed from the derivatives **NS-2**, **NS-8**, **NS-21**, and **NS-23** towards HT-29 cell line with IC_50_ between 4 and 19 μg/ml. More than 90% and 62% of the cells were found in the apoptotic phase on treatment with **NS-2** and **NS-21** respectively in comparison to the 68% for doxorubicin. Further, these derivatives arrested the cell growth in S and G2/M phase of the cell cycle.
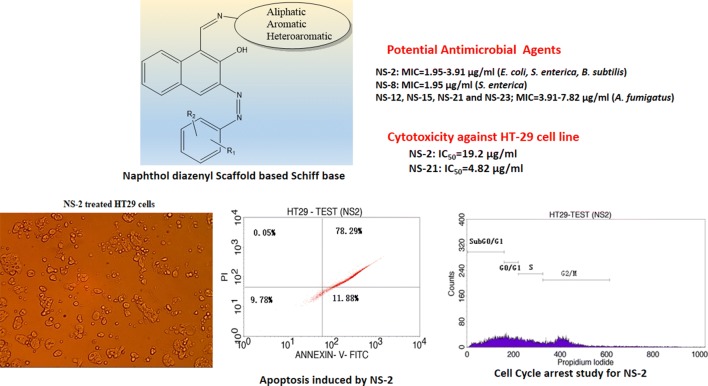

**Electronic supplementary material:**

The online version of this article (10.1186/s13065-019-0558-y) contains supplementary material, which is available to authorized users.

## Background

In spite of the development of new medicine, the cancer is still the leading cause of death worldwide and recognized as the uncontrolled and abnormal growth of the cells which is considered a multistep–multifaceted process involving a sequence of events and often accompanied with the suppression of immune system [1–3]. Patients with cancer are also at the increased risks of microbial infections as compared to the normal persons generally due to easy access of microorganisms as a result of interrupted epithelial barriers, compromised host defense, the absence of neutrophils, and shifts in the microbial flora [4–6]. Therefore, most patients diagnosed with cancer are also recommended with the antibiotics [7, 8].

Colorectal cancer (CRC), the second most common cancer in females and the third in males is a soft tissue neoplasm which arises from the lining of the large intestine (colon and rectum) [9–11]. The successful treatment of several malignancies including colorectal cancer is limited by lack of the complete eradication of the tumor cell population, the development of resistance to the chemotherapeutic agents probably through the modulation of anti-apoptotic or proliferative proteins of the survival cells and increased risk of microbial infections due to the suppression of host immune system [12–14]. For instance, *Escherichia coli* and *Salmonella* species have been reported as the possible cause of microbial infections in colorectal cancer [15–17]. The most commonly used therapeutic agents like oxaliplatin, cisplatin, fluoropyrimidines, irinotecan, in the treatment of colon cancer, have been shown to induce resistance in cancer cell killing resulting in the continued and rapid increase in the number of cancer cells [18, 19].

The induction of apoptosis as a result of DNA damage in cancer cells represents an effective strategy for preventing tumor growth [20]. The discovery of new molecules capable of reinstating the cellular mechanisms responsible for the induction of apoptosis in colon cancer cells and simultaneously having the potential to reduce the probability of microbial infections may provide additional benefits [21]. In the current research, we have planned the synthesis of novel hybridized molecules having cytotoxic and antimicrobial potential together.

Schiff’s bases have gained a lot of interest in the pharmaceutical and medicinal field in the past years [22]. They are the condensation products of carbonyl compounds with the primary amines having structural feature azomethine group (–HC=N–) substituted by various alkyl, aryl, cycloalkyl, or heteroaryl groups [23]. Schiff’s bases exhibit a broad spectrum of biological activities, comprising of antibacterial, antifungal, antiviral, antimalarial, anti-inflammatory and antipyretic properties [24]. Recently several reports have cited the potential of Schiff bases as cytotoxic agents [25–27]. Similarly, diazenyl compounds have also attracted the attention of researchers due to their extensive biological properties. Several diazenyl compounds (i.e. diazeniumdiolate prodrugs, diazenecarboxamides, diazenyl complexes etc.) have been already reported for their cytotoxic potential against different cancer cell lines in recent years [28–30]. These derivatives also reported having antimicrobial activity [31, 32]. The antimicrobial and cytotoxic effects of naphthol ring have already been disclosed [33, 34]. Hence, hybridization of the naphthol diazenyl (–N=N–) scaffold with the Schiff base (CH=N) can be a useful approach for the synthesis of new and effective compounds to act against both these diseases.

In this direction, we have synthesized novel naphthol diazenyl scaffold containing Schiff bases with various aromatic/heteroaromatic and aliphatic moieties and screened for their antimicrobial and cytotoxic potentials against human colorectal carcinoma cell line HT-29. The active agents were further evaluated for their apoptosis induction potential and cell cycle arrest studies. These dual-action novel derivatives with the advantage of cytotoxic potential against colon cancer and antimicrobial action from the same molecule may become highly desirable molecules therapeutically.

## Results and discussion

### Chemistry

**Fig. 1:**
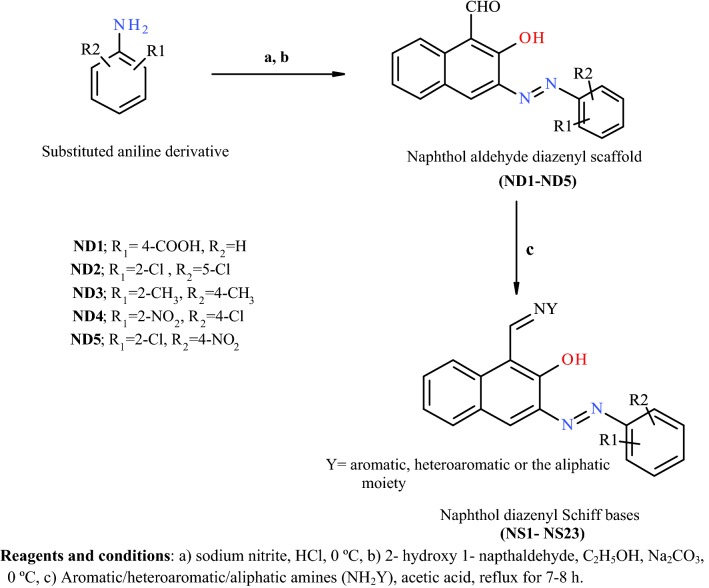
Synthetic methodology for naphthol diazenyl scaffold based Schiff bases

The synthetic scheme of naphthol diazenyl scaffold based Schiff bases is presented in Fig. 1. The different mono or di-substituted anilines in the presence of hydrochloric acid were diazotized with sodium nitrite, subsequently coupled with an ethanolic alkaline solution of 2-hydroxy naphthaldehyde to give azo dyes (**ND1**–**ND5**). The aldehyde group in naphthaldehyde azo dyes on reaction with different aromatic/heteroaromatic/aliphatic amines in the presence of catalytic amount of acetic acid resulted in **18** diazenyl Schiff bases (**NS-1** to **NS-23**) as given in Table 1. The structural confirmation of the target compounds was carried out by FTIR, UV–vis, NMR, mass spectroscopy, and elemental analysis. The thiophene substituted amines used in the reaction were prepared by the reported Gewald procedure [35]. The derivatives **NS-3**, **NS-17**, **NS-18**, **NS-19**, and **NS-20** have not been mentioned in the scheme as these derivatives did not meet the purity requirements for structural agreement by spectral techniques.

**Table 1:** Structure of various naphthol diazenyl based Schiff bases

S. No	Compound	R_1_	R_2_	Y
1	**NS-1**	4-COOH	H	
2	**NS-2**	4-COOH	H	
3	**NS-4**	4-COOH	H	
4	**NS-5**	2-Cl	5-Cl	
5	**NS-6**	2-Cl	5-Cl	
6	**NS-7**	2-CH_3_	4-CH_3_	
7	**NS-8**	2-Cl	5-Cl	
8	**NS-9**	2-Cl	5-Cl	
9	**NS-10**	2-Cl	5-Cl	
10	**NS-11**	2-Cl	5-Cl	
11	**NS-12**	2-Cl	5-Cl	
12	**NS-13**	4-COOH	H	
13	**NS-14**	2-NO_2_	4-Cl	
14	**NS-15**	2-NO_2_	4-Cl	
15	**NS-16**	2-Cl	4-NO_2_	
16	**NS-21**	2-NO_2_	4-Cl	
17	**NS-22**	2-Cl	5-Cl	
18	**NS-23**	4-COOH	H	

### UV spectroscopy

**Fig. 2:**
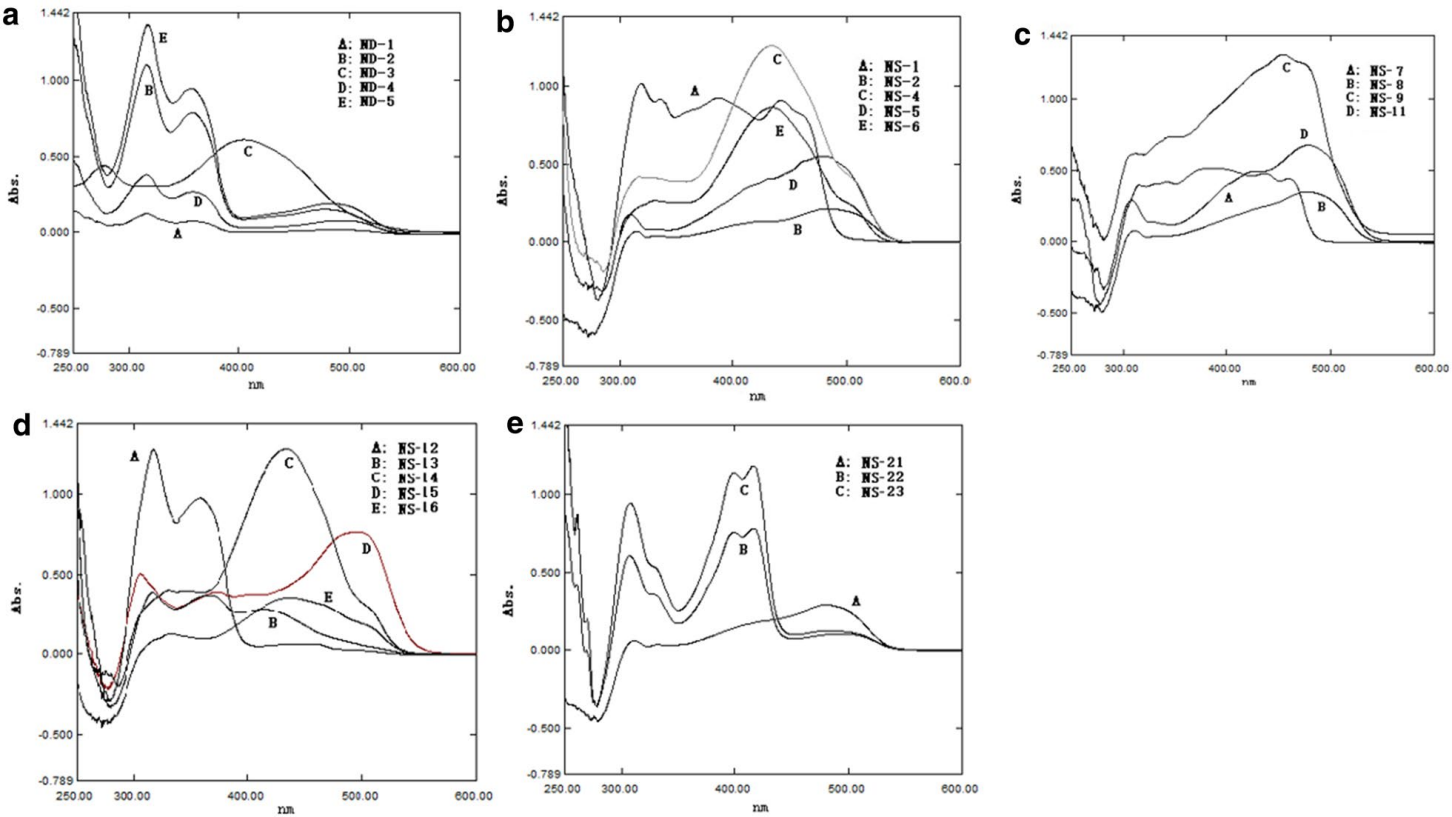
Electronic absorption spectra of dyes (**ND1**–**ND-5**) and naphthol diazenyl scaffold based Schiff bases (**NS1**–**NS-23**) in methanol at concentration of 1 × 10^−5^ M

The electronic absorption spectra of 2-hydroxy naphthaldehyde based dyes (**ND1**–**ND-5**) and diazenyl Schiff bases (**NS1**–**NS23**) were recorded in polar solvent methanol at the room temperature at the concentration of 1 × 10^−5^ M from the range of 200–800 nm. The scans and data have been presented in Fig. 2 and Table 2 respectively. The dyes (**ND1**–**ND5**) generally show absorption in the UV–visible range due to the presence of chromophore groups [36]. The absorption bands in the UV spectrum of dyes have been observed at 470–495 nm, 356–358 nm, 316 nm along with relatively minor bands in the range of 250–290 nm. The diazenyl Schiff bases have shown absorption maximum (λ_max_) at 430–497 nm, 340–397 nm, 330 nm, 305–315 nm and small bands in the range of 250–290 nm. The λ_max_ may be assigned to n–π* and π–π* transitions in the chromophoric –N=N– group, C=O group and other unsaturated groups present in the aromatic rings. The increased oxygenation on the ring generally results in bathochromic shifts. The dyes with nitro and carboxy groups substitution have shown absorption maximum at a higher wavelength as compared to the dyes with methyl group substitution. This may be attributed to the extended conjugated system due to the nitro and carboxyl groups.

**Table 2:** Absorption maximum (λ_max_) of dyes and naphthol diazenyl scaffold based Schiff bases

Comp.	Wavelength (λ_max_)	Comp.	Wavelength (λ_max_)	Comp.	Wavelength (λ_max_)	Comp.	Wavelength (λ_max_)
**ND-1**	482, 358, 316, 289	**NS-1**	442, 387, 335, 318	**NS-9**	453, 348, 312, 275, 272	**NS-16**	437, 333, 283, 265
**ND-2**	477, 357, 316, 254	**NS-2**	481, 340, 331, 315	**NS-10**	432, 335, 302, 275	**NS-21**	497, 416, 399, 307, 259
**ND-3**	404, 323, 279, 275	**NS-4**	434, 350, 328, 317, 280, 270	**NS-11**	477, 340, 329, 308,	**NS-22**	481, 339, 330, 312
**ND-4**	495, 358, 339, 316	**NS-5**	479, 331, 309, 270	**NS-12**	458, 434, 358, 317, 254	**NS-23**	482, 415, 399, 308, 269
**ND-5**	482, 357, 316	**NS-6**	434, 350, 340, 330, 280, 270	**NS-13**	414, 366, 316, 283		
–	–	**NS-7**	460, 436, 389, 341, 315, 254	**NS-14**	434, 350, 340, 330, 280		
–	–	**NS-8**	477, 340, 333, 311, 275, 260	**NS-15**	496, 403, 372, 306, 269		

### IR spectroscopy

The IR spectrum of synthesized compounds was determined by FTIR-attenuated reflectance (ATR) method. The dyes (**ND1**–**ND5**) exhibited C=O stretching vibration due to aldehyde group at 1630–1635 cm^−1^. The diazenyl Schiff bases exhibited –CH=N– absorption peak in the range of 1600–1636 cm^−1^. The C=O stretch due to the carboxyl group has been observed at 1675–1781 cm^−1^. The compounds having ester group exhibited another –C=O stretch at the 1721–1730 cm^−1^. The –C=C– a stretch of the aromatic rings appeared at 1565–1595 cm^−1^. The phenolic –OH group generally appeared as a broad peak in the range of 3650–3250 cm^−1^. The aldehydic =C–H group exhibited weak bands around 2850 cm^−1^ and 2750 cm^−1^. The presence of a band at 1465–1425 cm^−1^ confirmed the presence of azo linkage. The other peaks observed are the Ar–O stretching at 1100–1280 cm^−1^, –C=C– bending at 680–760 cm^−1^, the C–N stretching between 1000–1350 cm^−1^ and C–S stretching at 702 cm^−1^ and 617 cm^−1^. The aliphatic C–H stretch in methyl group was observed at 2850–3000 cm^−1^. The NO_2_ stretch confirmed by the two strong bands at 1280–1380 cm^−1^ and 1465–1520 cm^−1^. The bands in the range of 550–1050 cm^−1^ have been assigned to the C–X (halogen) absorption.

### NMR Spectroscopy

The ^1^H NMR and ^13^C NMR spectrum of the compounds were taken in CDCl_3_/DMSO solvents. The dyes (**ND1**–**ND5**) exhibited an aldehydic proton peak at *δ* 10.2–10.5 ppm. The Schiff bases exhibited a singlet at *δ* 8.5–9.8 ppm indicating the presence of CH=N proton with the complete disappearance of the peak at *δ* 10.2–10.5. The proton of the hydroxyl group on the 2nd position of the naphthalene ring generally appeared in the range of *δ* 12.5–16 ppm. The signals of the aromatic protons have been observed in the range of *δ* 6.8–8.5 ppm. The protons of the ethoxy group produced a classic triplet-quartet signal pattern at *δ* 1.30–1.49 ppm and 4.3–4.9 ppm respectively. The proton signal of the methylene group as in the case of **NS-2** and **NS-11** appeared as a singlet at 4.76–4.79 ppm. The furan ring presented three peaks as doublets at *δ* 6.23–6.37 ppm, 6.53–6.98 ppm, and 7.23–7.27 ppm respectively. The protons of the methylene groups of the aliphatic chain in **NS-21**, **NS-22**, and **NS-23** have been observed as the triplets at *δ* 1.90–2.13 ppm, *δ* 2.32–2.54 ppm, and *δ* 3.66–3.76 ppm respectively. The proton of the carboxyl group appeared in the range of *δ* 11–13 ppm. The protons of the saturated carbons of the cyclohexenyl ring appeared as two multiplets at *δ* 2.73–2.84 (4H, 2CH_2_) and *δ* 1.43–1.85 (4H, 2CH_2_). The carbon signals of the aromatic carbons in ^13^C NMR spectrum of Schiff bases observed between 109 and 156 ppm. The ^13^C NMR peaks at 165–177 ppm accounted for the carbonyl group. The carbon of the imine group was observed between 160 and 165 ppm. The ethoxy carbons appeared at the 60–63 ppm and 14–21 ppm respectively. The peak in the range of 54–57 ppm represented the methylene carbons of **NS-2** and **NS-11**. The carbon signals of saturated carbons of the cylohexenyl ring appeared in the range of 20–28 ppm. The ^1^H and ^13^C NMR spectra of most active compounds has been provided as the Additional file 1.

### Mass spectroscopy and CHNO/S analysis

The final confirmation of the synthesized compounds was done by mass spectroscopy. The diazenyl Schiff bases exhibited M^+^ (molecular ion peak) in positive chemical ionization mode. The % of C, O, N, H and S in the target compounds was within defined limits.

### Antimicrobial results

The synthesized derivatives **NS1** to **NS23** were evaluated for their antimicrobial potential in terms of minimum inhibitory concentration (MIC) and minimum bactericidal concentration (MBC)/minimum fungicidal concentration (MFC) values in µg/ml against standard drugs cefotaxime (antibacterial) and fluconazole (antifungal) and the results have been presented in Tables 3 and 4 respectively. Most of the synthesized derivatives had shown maximum activity against *E. coli* with MIC ranging from 1.95 to 31.25 µg/ml. These derivatives acted as bacteriostatic agents as well as bactericidal agents with MBC values ranging from 7.81 to 31.25 µg/ml. **NS-2** and **NS-8** had shown the highest activity against *S. enterica* (MIC = 1.95–3.91 µg/ml). **NS-2** was active against maximum bacterial strains but possessed very less activity against fungal strains whereas **NS-8** showed maximum bactericidal activity especially against *S. enterica* (MBC = 1.95 µg/ml). **NS-15** was most active against *E. coli* and *Aspergillus fumigatus* both. **NS-4**, **NS-5**, and **NS-7** were found active but against only *E. coli*. All synthesized derivatives have shown moderate activity against *Staphylococcus aureus*. The maximum activity was observed for the derivatives **NS-12**, **NS-21** and **NS-23** against *A.* *fumigatus* (MIC = 3.91–7.82 µg/ml and MBC = 3.91–15.62 µg/ml). The most of the synthesized derivatives found inactive against fungal strain *Aspergillus niger* with maximum activity shown by the derivative **NS-15** with the MIC of 15.62 µg/ml. From the above results, it is evident that the naphthol diazenyl scaffold has been essential for activity against *E. coli*. Mostly synthesized derivatives have shown maximum activity against *E. coli.* By the introduction of furfuryl ring at the naphthol diazenyl scaffold with carboxyl group substitution has dramatically increased the antibacterial activity. The presence of two thiophene rings in the molecule significantly increases the activity towards *S. enterica* (**NS-8**). Introduction of the aliphatic chain having three carbon atoms with a carboxyl group at the diazenyl scaffold expressively enhances the activity towards *A. fumigatus* which is decreased by substitution of two chloro groups at the diazenyl ring.

**Table 3:** Determination of MIC values (µg/ml) of naphthol diazenyl scaffold based Schiff bases

Compound	*E. coli*	*B. subtilis*	*S. enterica*	*S. aureus*	*A. niger*	*A. fumigatus*
MIC (µg/ml)						
**NS-1**	15.62	62.5	31.25	62.5	62.5	31.25
**NS-2**	3.91	3.91	1.95	31.25	125	125
**NS-4**	3.91	250	62.5	125	125	125
**NS-5**	7.81	62.5	31.25	125	125	125
**NS-6**	31.25	125	31.25	125	125	125
**NS-7**	3.91	250	62.5	62.5	125	125
**NS-8**	3.91	31.25	1.95	62.5	125	125
**NS-9**	15.62	125	62.5	62.5	125	125
**NS-10**	31.25	15.62	31.25	62.5	125	125
**NS-11**	15.62	125	31.25	125	125	62.5
**NS-12**	31.25	62.5	31.25	62.5	31.25	3.91
**NS-13**	3.91	15.62	62.5	62.5	31.25	15.62
**NS-14**	3.91	62.5	62.5	125	125	125
**NS-15**	1.95	62.5	62.5	62.5	15.62	3.91
**NS-16**	3.91	62.5	125	125	125	125
**NS-21**	15.62	31.25	62.5	125	62.5	3.91
**NS-22**	31.25	7.82	31.25	125	125	125
**NS-23**	15.62	62.5	31.25	62.5	62.5	7.82
**Cefotaxime**	3.91	3.91	1.95	31.25	–	–
**Fluconazole**	–	–	–	–	7.81	15.62

**Table 4:** Determination of MBC/MFC values (µg/ml) of naphthol scaffold based Schiff bases

Compound	*E. coli*	*B. subtilis*	*S. enterica*	*S. aureus*	*A. niger*	*A. fumigatus*
MBC/MFC (µg/ml)						
**NS-1**	31.25	125	62.5	62.5	62.5	62.5
**NS-2**	7.81	15.62	7.81	31.25	125	125
**NS-4**	31.25	250	12.55	125	125	125
**NS-5**	7.81	62.5	62.5	125	125	125
**NS-6**	62.5	125	62.5	125	125	125
**NS-7**	15.62	250	62.5	125	125	125
**NS-8**	15.62	31.25	1.95	62.5	125	125
**NS-9**	125	125	125	125	125	125
**NS-10**	125	31.25	125	125	125	125
**NS-11**	31.25	125	31.25	125	125	62.5
**NS-12**	31.25	62.5	62.5	62.5	31.25	7.81
**NS-13**	7.81	62.5	125	62.5	31.25	15.62
**NS-14**	7.81	62.5	62.5	125	125	125
**NS-15**	15.62	125	62.5	62.5	31.25	15.62
**NS-16**	7.81	125	125	125	125	125
**NS-21**	15.62	62.5	62.5	125	62.5	3.91
**NS-22**	31.25	125	125	125	125	125
**NS-23**	15.62	125	62.5	62.5	62.5	7.81
**Cefotaxime**	7.81	15.62	1.95	62.5	–	–
**Fluconazole**	–	–	–	–	15.62	31.25

The most active derivatives (**NS-1**, **NS-2**, **NS-4**, **NS-6**, **NS-8**, **NS-11**, **NS-12**, **NS-14**, **NS-15**, **NS-16**, **NS-21** and **NS-23**) based on the MIC and MBC values and based on the structural differences were further selected for cytotoxicity towards human colorectal carcinoma cell line (HT-29).

### Declines in HT-29 cell viability following exposure to test compounds

**Fig. 3:**
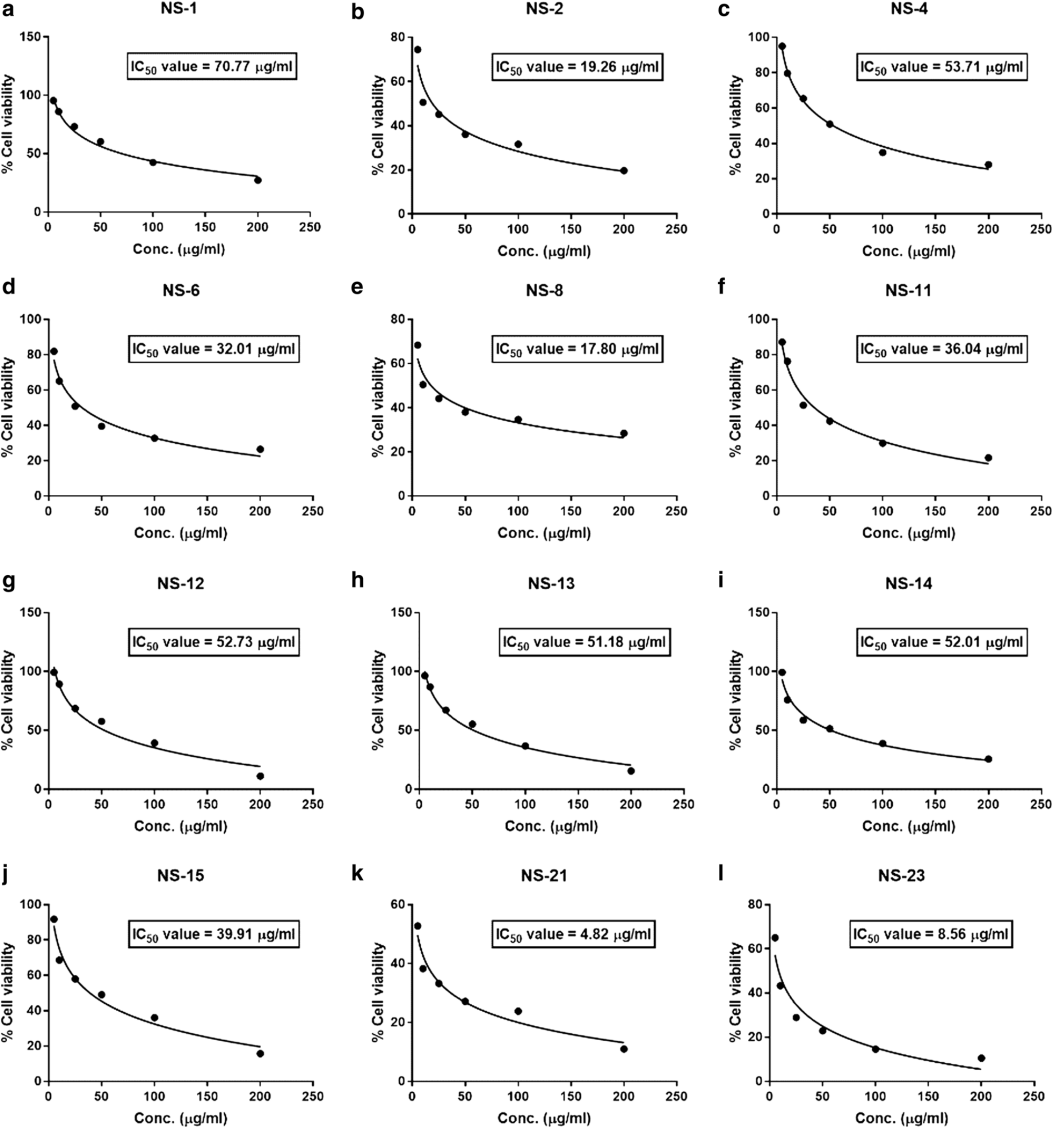
Survival curve plots for IC_50_ of synthesized naphthol based diazenyl Schiff bases: **a NS-1**; **b NS-2**; **c NS-4**; **d NS-6**; **e NS-8**; **f NS-11**; **g NS-12**; **h NS-13**; **i NS-14**; **j NS-15**; **k NS-21**; **l NS-23**

The cytotoxic potential of the most active derivatives (as mentioned in the above section) was evaluated by MTT (3,4,5-dimethylthiazol-2-yl)-2-5-diphenyltetrazolium bromide) assay which is based on the reduction of the yellow colored water-soluble tetrazolium dye MTT by the mitochondrial lactate dehydrogenase formed by the live cells to the formazan crystals, which display purple color upon dissolution into the suitable solvent. The intensity of the purple color is directly proportional to the number of viable cells and can be measured by spectrophotometer at 570 nm. The HT-29 cells were treated with different concentrations of these derivatives (5, 10, 25, 50, 100, 200 µg/ml) for 24 h and observed for cytotoxicity by MTT assay using ELISA reader. The cell survival plots were drawn between the % viability, and different concentrations of these test derivatives have been given in Fig. 3. The IC_50_ values were calculated from these plots. The observations in statistical data of cell cytotoxicity study suggest that the different test derivatives have decreased the cell viability in a dose-dependent manner. The % of cell viability decreased from 99 to 10% on treatment with the different concentrations of these test derivatives. At higher doses, the cell viability decreased up to 10–20% with almost all the test derivatives. Against HT-29 cells the selected test derivatives have shown good cytotoxic potential with the IC_50_ < 100 μg/ml (Table 5). The derivatives **NS-21** and **NS-23** both found to have very good cytotoxic potential against HT-29 cells with IC_50_ = 4–9 μg/ml as compared to the standard drug doxorubicin (IC_50_ = 5 μg/ml). **NS-2** and **NS-8** derivatives have also shown good cytotoxic potential with IC_50_ of 19.26 μg/ml and 17.8 μg/ml respectively followed by derivatives **NS-6**, **NS-11**, and **NS-15** which exhibited significant cytotoxicity with IC_50_ < 50 μg/ml. From the cytotoxicity study, the test derivative **NS-2** (being the best antibacterial agent) and **NS-21** (being the best cytotoxic agent having lowest IC_50_) were selected for the evaluation of apoptotic potential by flow cytometry.

**Table 5:** IC_50_ of synthesized naphthol based diazenyl Schiff bases against HT-29 cell line

S. No.	Sample code	IC_50_ (μg/ml)
1	NS-1	70.77
2	NS-2	19.26
3	NS-4	53.71
4	NS-6	32.01
5	NS-8	17.8
6	NS-11	36.04
7	NS-12	52.73
8	NS-13	51.18
9	NS-14	52.01
10	NS-15	38.91
11	NS-21	4.82
12	NS-23	8.56
13	Doxorubicin	5

### Morphological changes

**Fig. 4:**
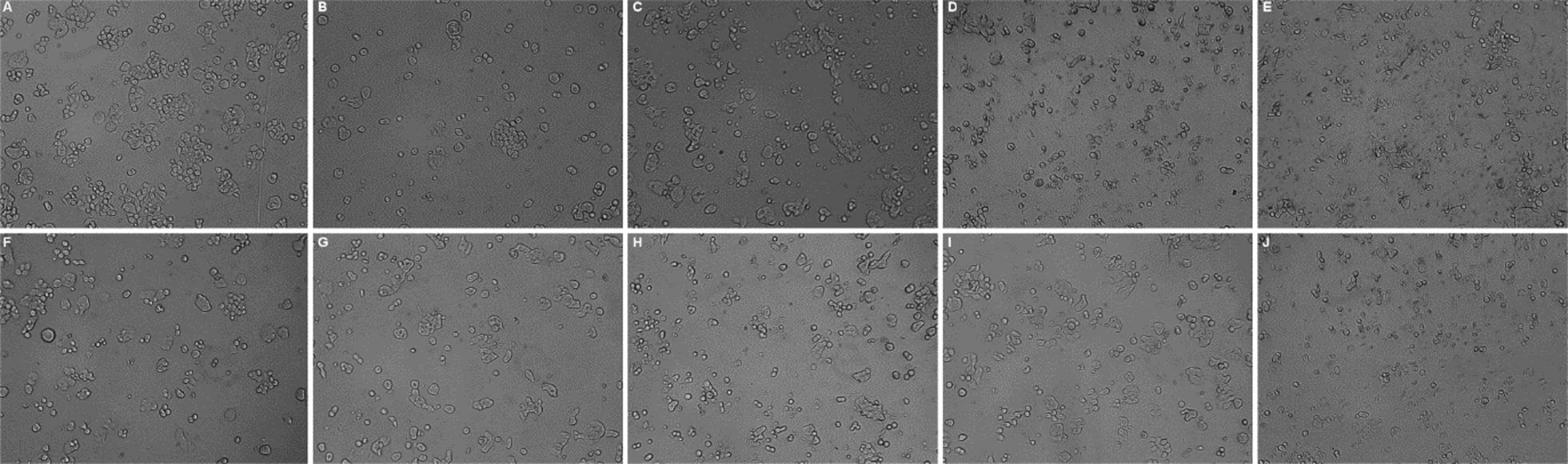
Morphological characterization of HT-29 cell after treatment with control, standard and different naphthol diazenyl scaffold Schiff bases recorded with the inverted phase microscope (Biolink) after 24 h of the treatment. **a** Untreated cells; **b** HT-29 cells treated with the standard at 5 µg/ml; **c** NS-2 treated cells at 25 µg/ml; **d** NS-4 treated cells at 50 µg/ml **d** NS-6 treated cells at 25 µg/ml; **e** NS-8 treated cells at 25 µg/ml; **f** NS-12 treated cells at 50 µg/ml; **g** NS-13 treated cells at 50 µg/ml; **h** NS-14 treated cells at 50 µg/ml; **i** NS-21 treated cells at 5 µg/ml; **j** NS-23 treated cells at 10 µg/ml

The HT-29 cells treated with different concentrations of the test derivatives were also observed under inverted phase microscope (Biolink) at 24 h for various morphological changes like the density of cells, the shape of the cells (HT 29 cells have the dual morphology of adherent as well as suspension nature), and any signs of shrinkage. In Fig. 4, it is evident that the test derivatives have reduced the number and clumping of cells. The higher concentrations of the test derivatives have significantly reduced the number of HT-29 cells.

### Apoptosis induction by test compounds

**Fig. 5:**
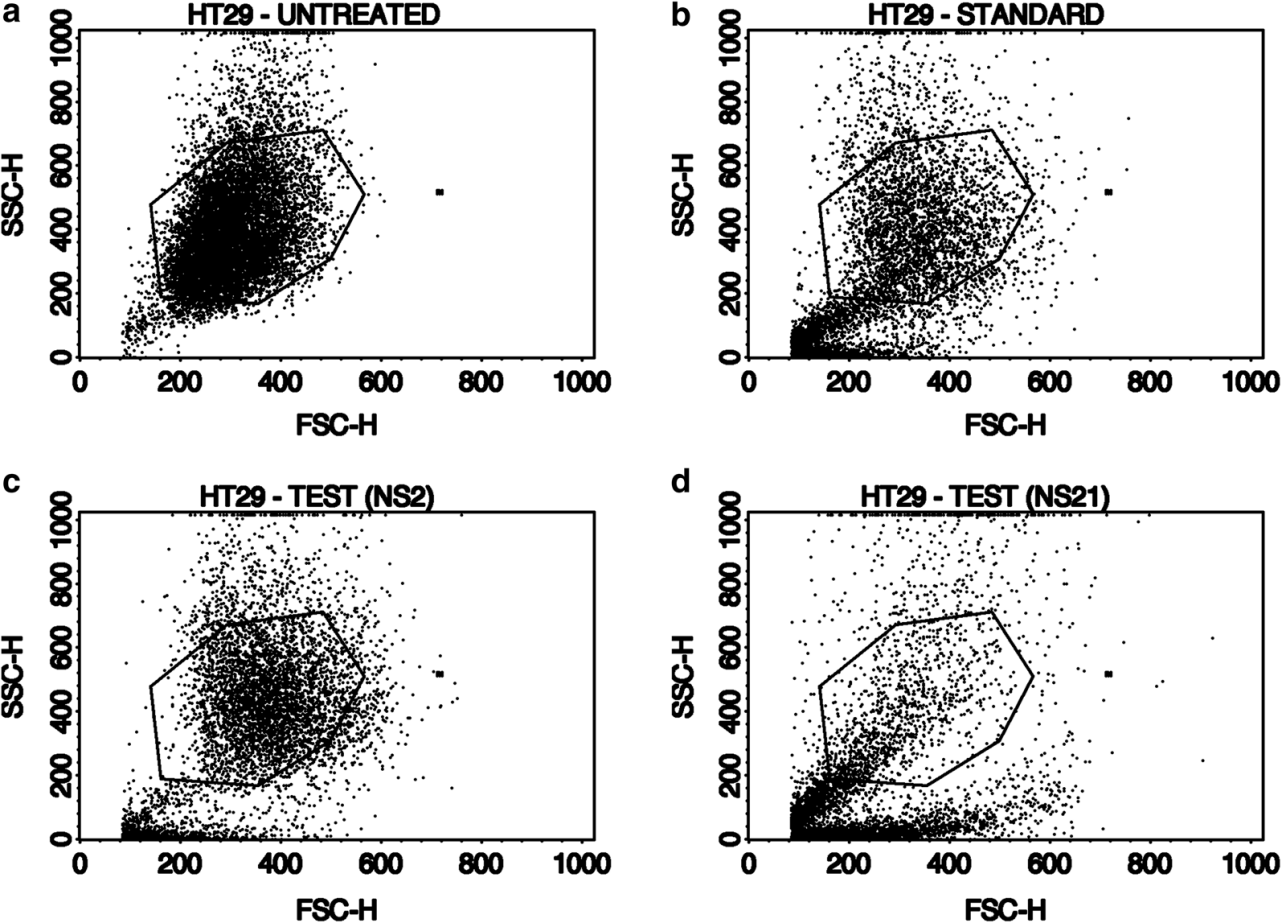
Dot plot (**a** untreated; **b** standard;  **c NS-2**; **d** **NS-21**) which refers all the cells took for the analysis and selected some cells in the centre of this plot called as gated cells (selected region) R1-Region 1 since those cells are single cells and cells of our interest. Above the gated region cells are clumped cells or may be double or triple clump of cells and below the gated region cells are debris or dead cells. Cells based on the cells size and granularity will appear in the dot plot where FSC—Forward scattering light refers granularity and shape of cells and SSC—Side Scattering light refers size of the cells

**Fig. 6:**
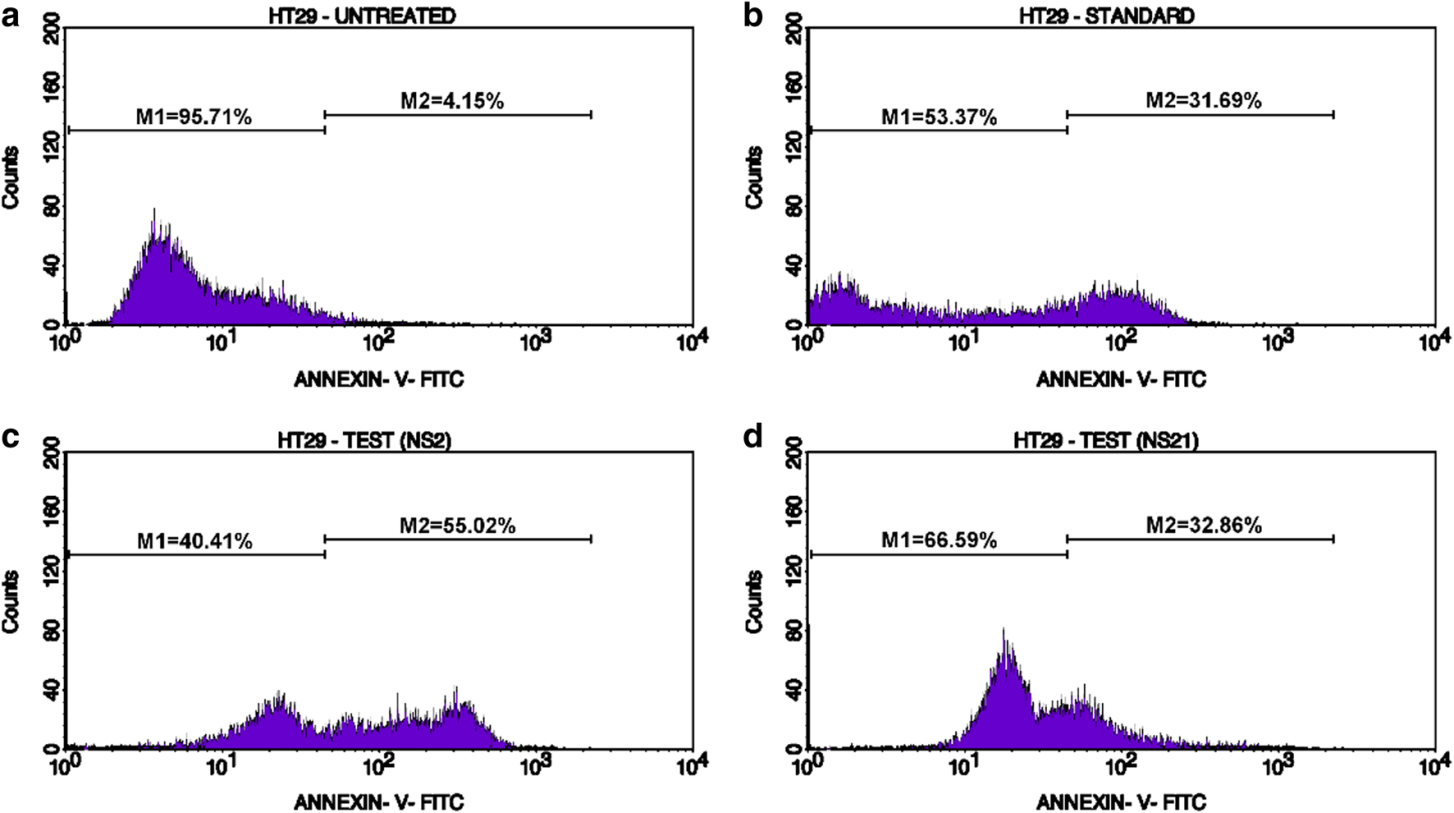
Histogram presenting the no. of viable and apoptotic cells in **a** untreated; **b** standard;  **c** **NS-2**; **d** **NS-21** on HT-29 cells. The M1 populations are the viable cells that are low in mean Annexin V-FITC fluorescence intensity while M2 populations are the apoptotic cells that have taken up the Annexin V-FITC and are high in mean Annexin V-FITC fluorescence intensity

**Fig. 7:**
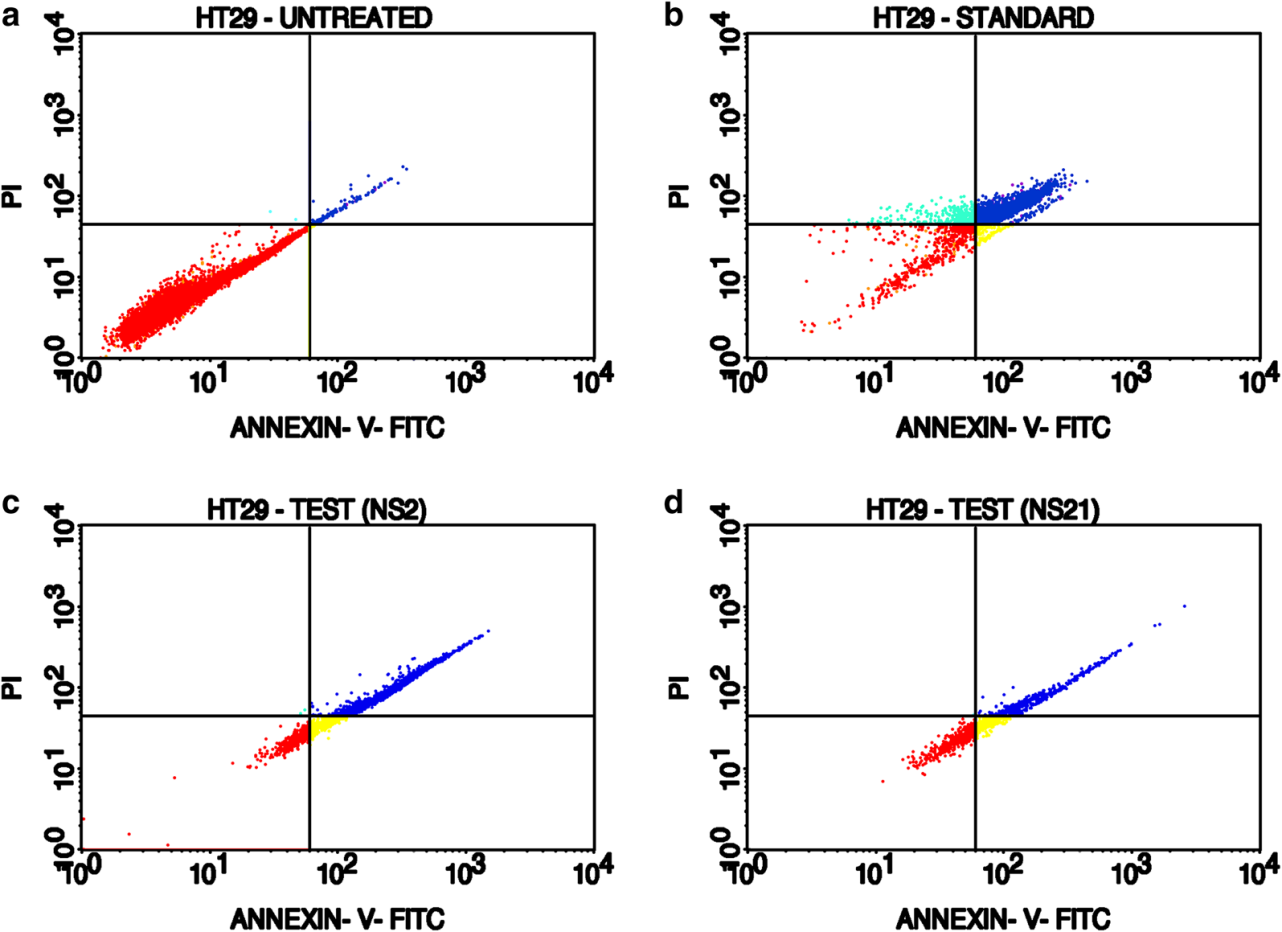
Annexin V-PI expression study of untreated, standard and test compounds (**NS-2** and **NS-21**) on HT-29 cells using BD FACScalibur, Cell Quest Pro Software (Version: 6.0). Quadrants showing the expression % of various type of cells: **a** lower left (LL) quadrant represent the % of viable cells, **b** lower right (LR) quadrant represent the % of early apoptotic cells, **c** upper left (UL) quadrant represent the % of necrotic cells, **d** upper right (UR) quadrant represent the % of late apoptotic cells against the Annexin V-FITC and propidium iodide Stain. Where Annexin V-FITC—primary marker, PI—propidium iodide (secondary fluorescence marker)

**Fig. 8:**
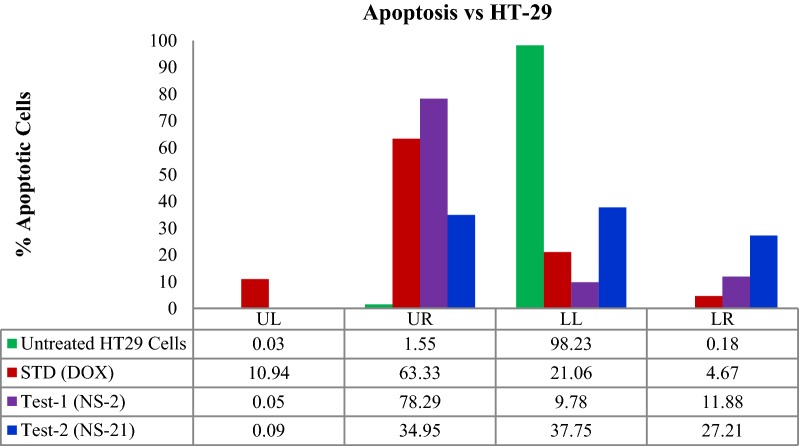
Comparison of control, standard and test compounds (**NS-2** and **NS-21**) showing the % of viable, early apoptotic, late apoptotic and necrotic cells against the Annexin V-FITC and Propidium iodide stain assay

The induction of apoptosis in HT-29 cells was studied by the Annexin-V (AV)/propidium iodide (PI) double staining assay using flow cytometry. This assay is based on the interaction of AV with phosphatidylserine (PS) (normally present in the inner membrane but translocated to the outer membrane during apoptosis) on the cell surface. The strong affinity of AV-FITC with PS due to loss of plasma membrane asymmetry leads to AV+/PI− staining in the early apoptotic cells. The intact cell membrane of the live cells is not permeable to AV and PI and hence represents AV−/PI− staining whereas AV+/PI+ staining represents the late apoptotic cells. Necrotic cells are represented by AV−/PI+ staining on account of penetration of PI through the membranes and intercalation into the nucleic acid due to loss of the plasma and nuclear membrane integrity. The HT-29 cells were treated with the test derivatives and standard drug (doxorubicin) at their IC_50_ concentrations (**NS-2**: IC_50_ = 19.6 μg/ml, **NS-21**: IC_50_ = 4.82 μg/ml, Doxorubicin: IC_50_ = 5.0 μg/ml) for 24 h and then analyzed by flow cytometry [BD FACScalibur, Cell Quest Pro Software (Version: 6.0)]. Figures 5, 6, 7, 8, represents the cytometry results for the test derivatives vs untreated control and doxorubicin. Figure 5a–d indicates the selection of the cells which are mainly single and segregated required for the cell cytometry. Figure 6a–d represents the histogram of AV-FITC vs cell counts, detecting the number of AV-FITC positive cells. Figure 7a reveals the untreated HT-29 cells representing the 98.23% population of the viable cells, 0.03% necrotic cells, 0.18**%** early apoptotic, and 1.55% late apoptotic/secondary necrotic cells. Figure 7b corresponds to the doxorubicin-treated HT-29 cells with 21.06% population of viable cells, 10.95% necrotic cells, 4.67% of early apoptotic cells and the 63.33% of the late apoptotic cells. In case of **NS-2** and **NS-21** (Fig. 7c, d), the HT-29 cells population were; 9.78% and 37.75% viable population, 0.05% and 0.09% necrotic cells, 11.88% and 27.21% early apoptotic cells, and 78.29% and 34.95%, late apoptotic cells respectively. These results indicate that the cells treated with test derivatives **NS-2** and **NS-21** have significantly undergone apoptosis after 24 h post-treatment compared to the untreated cells and doxorubicin-treated cells. The **NS-2** has induced apoptosis in > 90% of the cells in the early and late apoptotic phase, whereas the **NS-21** has shown comparable results (62% of the cells in the early and late apoptotic phase) to that of the doxorubicin (68% of apoptotic cells) (Fig. 8). Additionally, the treatment of cells with the **NS-2** and **NS-21** demonstrated no concurrent increase in the number of necrotic cells as in the case of doxorubicin which showed an increase in necrotic cells up to 10.89%. Therefore, the **NS-2** and **NS-21** attest the antiproliferation mechanism in HT-29 cells through the induction of apoptosis.

### S and G2/M phase arrest of the cell cycle

**Fig. 9:**
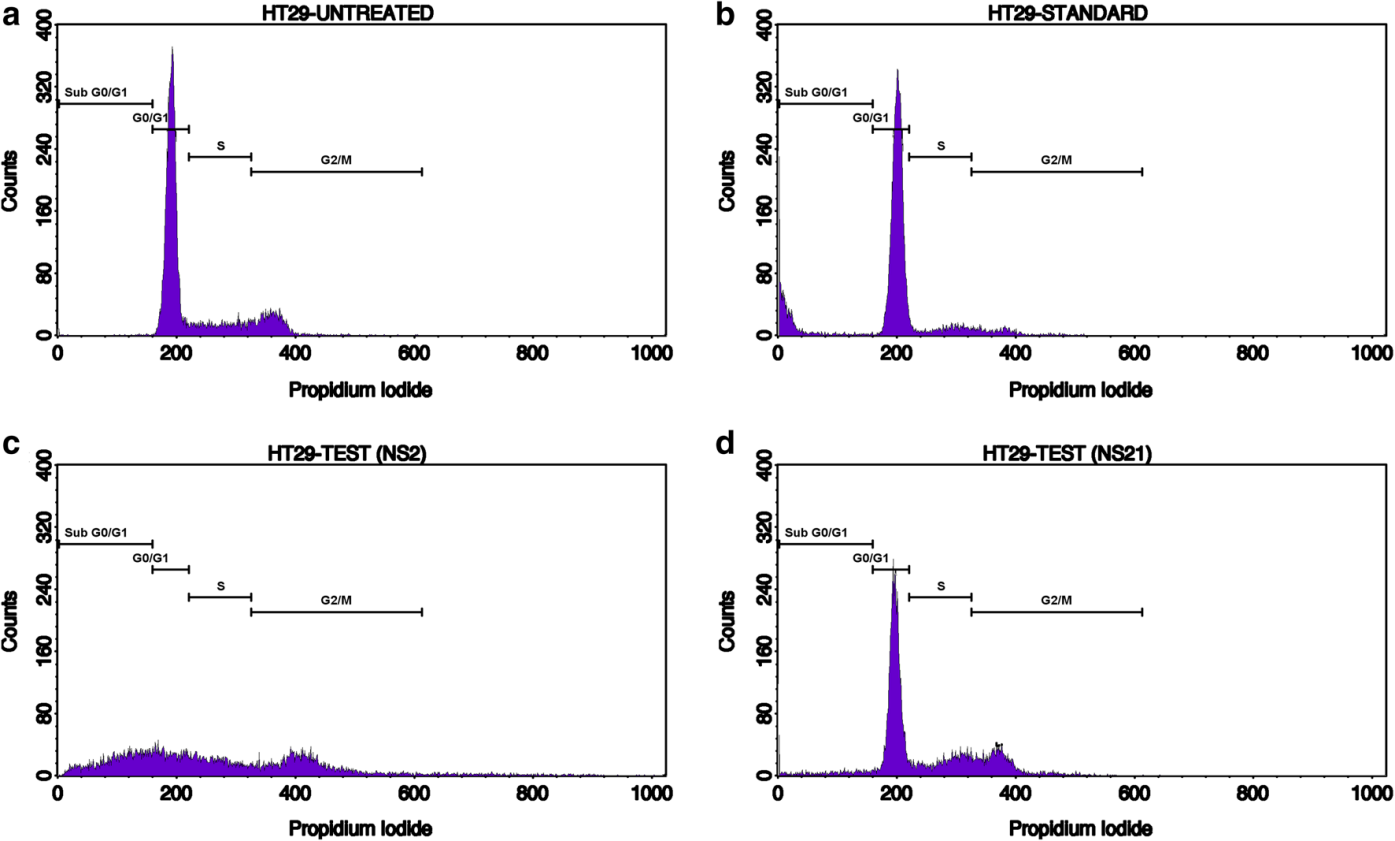
Histograms showing the cell cycle distribution of untreated (**a**), standrad drug (doxorubicin-5 μg) (**b**), and test compound-1 (**NS-2**) (**c**) and test compound-2 (**NS-21**) (**d**) against HT29 cells using BD FACScalibur. PI histogram of the gated cell singlets distinguishes cells at the Sub G0/G1, G0/G1, S, and G2/M cycle phases. Gating of cell cycle phases is approximate and can be refined using software (Cell Quest Software, Version 6.0) analysis. For each analysis 10,000 singlet cells were gated into Sub G0/G1, G0/G1, S and G2/M phases for the analysis for all samples including controls as indicated in the histograms

**Fig. 10:**
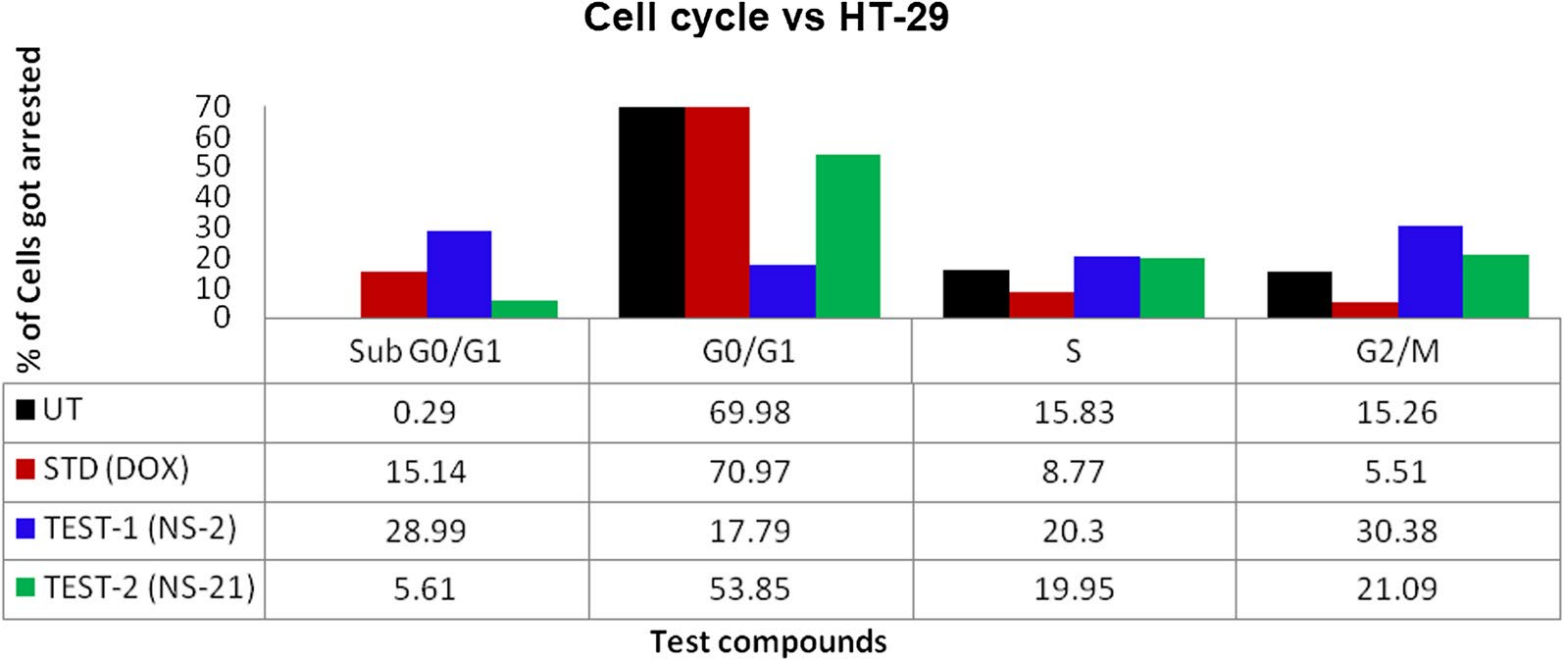
Comparison of control, standard and test compounds (**NS-2** and **NS-21**) showing the % of cell arrested in different phases of the cell cycle

To determine the possible effects on arresting the specific phase of cell growth, the HT-29 cells were treated with the IC_50_ concentrations of the **NS-2**, **NS-21**, and doxorubicin for 24 h and then stained with PI and observed for the count of cells in each phase of the cell cycle by flow cytometry. The results of the study have been presented in Figs. 9a–d and 10. Figure 9a represents the untreated cells with a population of 0.29% in the sub-G0/G1 phase, 69.98% in G0/G1 phase, 15.83% in S phase, and 15.26% in G2/M phase of the cell cycle. The untreated cells or control presented very less number of the cells in the sub-G0/G1 phase a condition of a very sparse population of the apoptotic cells. On the contrary, doxorubicin treated cells presented a population of 15.14% cells in the sub-G0/G1 phase, which constituted a large proportion of the apoptosis cells (Fig. 9b). The doxorubicin has arrested the cell cycle in G0/G1 phase, and the number of cells in this phase is 70.97% with a significant decrease in the proportion of cells in S (8.77%) and the G2/M (5.21%) phases as shown in Fig. 10. The test derivatives **NS-2** and **NS-21** presented the 28.29% and 5.16% of the cell population in the sub-G0/G1 phase respectively as indicated in Fig. 9c, d. The increased sub G0/G1 peak in the cell cycle for **NS-2** treated HT-29 cells demonstrate enhanced apoptosis in these cells. The derivative **NS-2** also showed a dramatic decrease in the proportion of the G0/G1 phase cells (17.79%) as compared to the control group cells (69.98%) and arrested the cell growth in the S phase (20.3% cells population) and G2/M phase (30.38% cells population) of the cell cycle (Figs. 9c and 10). The derivative **NS-21** have also followed the similar trend of arresting cells in the S phase (19.95%) and G2/M phase (21.09%) of the cell cycle. From the above results, it is clear that the derivative NS-2 has high ability to induce apoptosis in HT-29 cells in comparison to the doxorubicin. Both these test derivatives have arrested the cell cycle in the S and G2/M phases of the cell cycle, which is crucial for the cell division and proliferation.

## Experimental

The chemicals and other reagents for synthesis were procured from Merck Chemicals (India) and used without further purification. The nutrient media for the antimicrobial evaluation and other chemicals required for cytotoxicity study were purchased from Hi-Media Laboratories (India). The microbial strains were acquired from Institute of Microbial Technology and Genebank (IMTECH), Chandigarh. The FTIR spectrophotometer Bruker 12060280 was used for recording the IR spectra. Electronic absorption spectra were taken in the methanolic solution of diazenyl Schiff bases on double beam UV–visible spectrophotometer (Shimadzu). The purity of compounds was checked by NMR spectroscopy (^1^H NMR and ^13^C NMR), carried out in deuterated CDCl_3_ and DMSO solvents on Bruker Avance II 300 NMR spectrometer at a frequency of 300 MHz and 75 MHz and Bruker Avance II 400 NMR spectrometer at a frequency of 400 MHz and 100 MHz respectively. The elemental analysis was performed on CHNN/CHNS/O analyzer (Flash\EA1112Nseries, Thermofinnigan, Italy). The structures of the synthesized derivatives were confirmed by mass spectra, taken on the Advion expression CMS, USA mass spectrometer with APCI mode as the ion source.

### General procedure for the synthesis of diazenyl Schiff bases (NS1-NS23)

Hydrochloric acid (8 ml, 33%) was added dropwise to the well-stirred suspension of mono- or di-substituted aniline derivative (0.01 mol) in H_2_O (15 ml) followed by cooling to 0–5 °C on an ice bath. Afterward, a cold solution of sodium nitrite (0.01 mol) in H_2_O (7 ml) was added with continuous stirring throughout 5–10 min. The stirring was further continued for 30 min at 0–5 °C. The excess of nitrous acid was neutralized by the addition of urea and tested by potassium iodide paper. The clear diazo solution formed was used for successive coupling reaction and added dropwise to the well-stirred solution of 2-hydroxy-1-naphthaldehyde (0.01 mol) in ethanol, over a period of 10–15 min at 0–5 °C. The pH of the solution was maintained at 8.5 by simultaneous addition of Na_2_CO_3_ solution (10% w/v) with continuous stirring, maintaining the temperature below 5 °C. The solution was acidified with HCl (pH = 1.0) at the completion of the reaction to precipitate the azo dyes (**ND1**–**ND5**) which were filtered, washed with NaCl solution (5% w/v), and air dried. The dyes (**ND1**–**ND5**) were further used for the synthesis of Schiff bases (**NS1**–**NS23**) by reaction of diazenyl dyes (5 mmol) with various aliphatic, aromatic or heteroaromatic amines (5 mmol) in ethanol/DMSO solvents and traces (5–7 drops) of acetic acid. The refluxing of the reaction mixture was continued for 7–8 h until the reaction completion was confirmed by TLC. The reaction volume was concentrated to half and kept at 10–15 °C for the precipitation of Schiff bases [37]. The precipitated Schiff bases were collected by filtration, washed with ice-cold ethanol and dried in air. The synthesized derivatives were purified by column chromatography and recrystallization techniques.

### Analytical data

#### 4-((4-Formyl-3-hydroxynaphthalen-2-yl)diazenyl)benzoic acid (ND-1)

MF: C_18_H_12_N_2_O_4_; Orange color; Yield: 80%; R_f_ = 0.31 (hexane/ethyl acetate 5:2); mp: 123–125 °C; IR (ATR, cm^−1^) ν_max_: 3398.03, 3302.80, 3246.47, 3055.65, 2979.11, 2822.97, 1691.70, 1628.16, 1512.03, 1460.32, 1396.83, 1306.56, 1242.68, 1159.08, 1074.38, 953.15, 858.47, 792.13, 744.46, 653.00; ^1^H NMR (400 MHz, DMSO-d_6_) δ: 14.19 (s, 1H), 12.93 (s, 1H), 10.78 (s, 1H), 8.97 (d, *J *= 5.6 Hz, 1H), 7.95–8.07 (m, 2H), 7.47–7.97 (m, 4H), 7.06–7.39 (m, 2H); ^13^C NMR (100 MHz, DMSO-d_6_) δ: 193.34, 171.56, 164.39, 157.31, 142.20, 140.01, 138.89, 132.18, 129.77, 128.05, 124.71, 122.64, 119.21, 112.92.

#### 3-((2,5-Dichlorophenyl)diazenyl)-2-hydroxy-1-naphthaldehyde (ND-2)

MF: C_17_H_10_Cl_2_N_2_O_2_; Orange color, Yield: 85%; R_f_ = 0.34 (hexane/ethyl acetate 5:2); mp: 58–60 °C; IR (ATR, cm^−1^) ν_max_: 3365.00, 3280.23, 3206.33, 3021.98, 1630.08, 1553.22, 1460.76, 1397.38, 1305.59, 1241.64, 1160.01, 1086.20, 1024.67, 967.95, 857.79, 789.12, 742.15, 648.73; ^1^H NMR (400 MHz, DMSO-d_6_) δ: 12.01 (s, 1H), 10.84 (s, 1H), 8.94 (s, 1H), 8.14 (s, 1H), 7.89 (d, *J *= 8.0 Hz, 1H), 7.62 (t, *J *= 8.0 Hz, 1H), 7.42 (t, *J *= 8.0 Hz, 1H), 7.29 (d, *J *= 8.0 Hz, 1H), 7.02–7.24 (m, 2H); ^13^C NMR (100 MHz, DMSO-d_6_) δ: 193.20, 164.45, 156.14, 141.16, 140.01, 137.23, 134.26, 132.13, 129.64, 128.32, 124.45, 123.14, 118.23, 113.65.

#### 3-((2,4-Dimethylphenyl)diazenyl)-2-hydroxy-1-naphthaldehyde (ND-3)

MF: C_19_H_16_N_2_O_2_; Orange color; Yield: 69%; R_f_ = 0.39 (hexane/ethyl acetate 5:2); mp: 102–104 °C. IR (ATR, cm^−1^) ν_max_: 3489.40, 3055.30, 1734.34, 1631.55, 1596.00, 1471.20, 1433.55, 1391.80, 1348.90, 1241.81, 1144.27, 795.81, 736.98, 655.26; ^1^H NMR (400 MHz, DMSO-d_6_) δ: 13.84 (s, 1H), 10.84 (s, 1H), 8.94 (s, 1H), 8.15 (s, 1H), 7.86 (d, *J *= 8.0 Hz, 1H), 7.63 (t, *J *= 8.0 Hz, 1H), 7.40 (t, *J *= 8.0 Hz, 1H), 7.02–7.38 (m, 3H), 2.35 (s, 3H), 2.38 (s, 3H); ^13^C NMR (100 MHz, DMSO-d_6_) δ: 194.34, 165.46, 156.22, 141.16, 140.01, 139.13, 135.26, 133.23, 132.13, 129.64, 128.16, 124.45, 123.45, 118.23, 21.23, 18.17.

#### 3-((4-Chloro-2-nitrophenyl)diazenyl)-2-hydroxy-1-naphthaldehyde (ND-4)

MF: C_17_H_10_ClN_3_O_4_; Orange color; Yield: 72%; mp: 88–90 °C; R_f_ = 0.37 (hexane/ethyl acetate 5:2); IR (ATR, cm^−1^) ν_max_: 3409.00, 3067.45, 2915.67, 1738.45, 1635.15, 1590.59, 1464.22, 1398.95, 1311.40, 1246.47, 1163.39, 1079.34, 857.43, 793.83, 744.81, 529.16, 468.11; ^1^H NMR (400 MHz, DMSO-d_6_) δ: 14.84 (s, 1H), 10.81 (s, 1H), 8.54 (s, 1H), 8.05 (s, 1H), 7.89 (d, *J *= 8.0 Hz, 1H), 7.62 (t, *J *= 8.0 Hz, 1H), 7.40 (t, *J *= 8.0 Hz, 1H), 7.12–7.38 (m, 3H); ^13^C NMR (100 MHz, DMSO-d_6_) δ: 193.01, 168.12, 163.20, 154.16, 142.11, 140.02, 135.81, 132.23, 130.29, 129.65, 128.11, 127.56, 126.34, 123.12, 116.71.

#### 3-((2-Chloro-4-nitrophenyl)diazenyl)-2-hydroxy-1-naphthaldehyde (ND-5)

MF: C_17_H_10_ClN_3_O_4_; Orange color; Yield: 76%; mp: 95–97 °C; R_f_ = 0.32 (hexane/ethyl acetate 5:2); IR (ATR, cm^−1^) ν_max_: 3409.00, 3067.45, 2915.67, 1738.45, 1635.15, 1590.59, 1464.22, 1398.95, 1311.40, 1246.47, 1163.39, 1079.34, 857.43, 793.83, 744.81, 529.16, 468.11; ^1^H NMR (400 MHz, DMSO-d_6_) δ: 14.62 (s, 1H), 10.65 (s, 1H), 8.57 (m, 1H), 8.14 (s, 1H), 7.99 (d, *J *= 7.6 Hz, 1H), 7.76 (d, *J *= 7.6 Hz, 1H), 7.64–7.69 (m, 1H), 7.51 (t, *J *= 8.0 Hz, 1H), 7.37 (d, *J *= 8.4 Hz, 1H), 6.82 (d, *J *= 9.2 Hz, 1H); ^13^C NMR (100 MHz, DMSO-d_6_) δ: 193.21, 167.39, 161.10, 155.21, 143.25, 140.01, 134.08, 132.00, 130.26, 129.75, 127.72, 127.27, 125.56, 122.74, 116.71.

#### 2-Hydroxy-4-((2-hydroxy3-(4-carboxyphenyldiazenyl)naphthalen-1-yl)methyleneamino) benzoic acid (NS-1)

Maroon color, Yield: 61%; mp: 273–275 °C; R_f_ = 0.72 (hexane/ethyl acetate 3:2); IR (ATR, cm^−1^) ν_max_: 3682.17, 3555.32, 3497.91, 3384.31, 3324.28, 1781.17, 1744.65, 1619.56, 1524.89, 1425.25, 1330.14, 1277.93, 1201.80, 1132.58, 1024.97, 947.13, 725.95, 627.24; ^1^H NMR (400 MHz, DMSO-d_6_) δ: 15.78 (s, 1H), 12.03–12.24 (m, 2H), 10.82 (s, 1H), 9.71 (s, 1H), 8.92 (d, *J *= 4.8 Hz, 1H), 8.54 (d, *J *= 8.4 Hz, 1H), 7.82–8.12 (m, 5H), 7.56 (t, *J *= 7.2 Hz, 1H), 7.37 (t, *J *= 7.2 Hz, 1H), 7.08–7.10 (m, 2H); ^13^C NMR (100 MHz, DMSO-d_6_) δ: 170.56, 168.66, 165.54, 160.40, 154.69, 142.31, 139.29, 137.19, 136.30, 133.36, 129.40, 128.38, 127.37, 123.91, 123.06, 121.55, 121.09, 118.74, 116.35, 108.32; APCI-MS m/z found for C_25_H_17_N_3_O_6_: 455 (M^+^); Anal. calcd for C_25_H_17_N_3_O_6_: C 65.93, H 3.76 N 9.23, O 21.08 found: C 65.92, H 3.75, N 9.24, O 21.07.

#### 4-((4-((Furan-2-ylmethylimino)methyl)-3-hydroxynaphthalen-2-yl)diazenyl)benzoic acid (NS-2)

Maroon color, Yield: 66%; mp: 283–285 °C; R_f_ = 0.52 (hexane/ethyl acetate 5:2); IR (ATR, cm^−1^) ν_max_: 3400.17, 1732.62, 1702.98, 1636.96, 1547.93, 1506.09, 1465.93, 1424.45, 1395.38, 1317.10, 1260.22, 1215.21, 1161.60, 1038.68, 1009.89, 967.34, 902.84, 856.15, 822.68, 748.38, 673.59, 654.15, 635.64; ^1^H NMR (300 MHz, CDCl_3_) δ: 14.20 (s, 1H), 12.99 (s, 1H), 8.92 (s, 1H), 7.91 (d, *J *= 8.1 Hz, 2H), 7.59–7.90 (m, 2H), 7.37–7.56 (m, 5H), 7.27 (d, *J *= 6.6 Hz, 1H), 6.98 (d, *J *= 6.6 Hz, 1H), 6.37 (d, *J *= 6.6 Hz, 1H), 4.79 (s, 2H); ^13^C NMR (75 MHz, CDCl_3_) δ: 170.19, 165.15, 161.10, 156.13, 151.34, 142.29, 141.27, 134.16, 133.13, 130.80, 129.47, 128.95, 128.46, 126.98, 126.09, 125.83, 123.28, 121.48, 116.27, 108.41, 56.41; APCI-MS m/z found for C_23_H_17_N_3_O_4_: 399.12 (M^+^); Anal. calcd for C_23_H_17_N_3_O_4_: C 69.17, H 4.29, N 10.52, O 16.02 found: C 69.19, H 4.26, N 10.49, O 16.05.

#### 4-((4-((3-(Ethoxycarbonyl)-4,5,6,7-tetrahydrobenzo[*b*]thiophen-2-ylimino)methyl)-3-hydroxynaphthalen-2-yl)diazenyl)benzoic acid (NS-4)

Maroon crystals; Yield: 71%; mp: 130–132 °C; R_f_ = 0.57 (hexane/ethyl acetate 7:3); IR (ATR, cm^−1^) ν_max_: 3430.05, 3368.85, 3255.36, 3070.81, 2886.96, 1816.95, 1673.57, 1599.99, 1545.80, 1454.97, 1356.37, 1281.25, 1225.93, 1119.51, 1069.00, 933.52, 893.52, 814.12, 725.42, 624.59; ^1^H NMR (400 MHz, DMSO-d_6_) δ:14.49 (s, 1H), 12.99 (s, 1H), 9.46 (s, 1H), 8.45 (d, *J *= 8.8 Hz,1H), 8.02–8.07 (m, 2H), 7.87–7.98 (m, 2H), 7.59–7.62 (m, 1H), 7.41–7.50 (m, 1H), 7.19 (d, *J *= 8.8 Hz, 1H), 6.80 (d, *J *= 8.8 Hz, 1H), 4.32 (q, *J *= 7.2 Hz, 2H), 2.73–2.75 (m, 4H), 1.79–1.80 (m, 4H), 1.35 (t, *J *= 7.2 Hz, 3H); ^13^C NMR (75 MHz, CDCl_3_) δ: 172.03, 165.30, 163.53, 153.75, 153.17, 138.21, 135.90, 132.67, 130.80, 129.31, 127.94, 127.77, 123.71, 123.24, 120.67, 119.43, 109.66, 60.77, 26.33, 25.56, 22.89, 22.54, 14.45; APCI-MS m/z found for C_29_H_25_N_3_O_5_S: 527.29 (M^+^); Anal. calcd for C_29_H_25_N_3_O_5_S: C 66.02, H 4.78, N 7.96, O 15.16, S 6.08 found C 66.04, H 4.76, N 7.93, O 15.14.

#### 1-((4-Chloro-2-nitrophenylimino)methyl)-3-((2,5-dichlorophenyl)diazenyl)naphthalen-2-ol (NS-5)

Orange color, Yield: 67%; mp: 163–165 °C; R_f_ = 0.46 (hexane/ethyl acetate 5:2); IR (ATR, cm^−1^) ν_max_: 3327.59, 3123.45, 3036.46, 1705.79, 1636.95, 1554.55, 1505.69, 1460.28, 1407.17, 1347.62, 1251.66, 1191.63, 1122.96, 1072.17, 992.62, 919.62, 830.94, 801.99, 743.56, 681.44, 648.12, 628.84; ^1^H NMR (400 MHz, DMSO-d_6_) δ: 14.82 (s, 1H), 9.81 (s, 1H), 8.54 (s, 1H), 8.14–8.35 (m, 2H), 7.86–7.99 (m, 2H), 7.43–7.64 (m, 3H), 7.02–7.40 (m, 3H); ^13^C NMR (100 MHz, DMSO-d_6_) δ: 166.19, 156.19, 148.38, 133.66, 133.59, 129.58, 129.53, 129.49, 129.40, 129.19, 129.11, 128.47, 127.31, 123.10, 124. 64, 122.89, 119.89, 109.71; APCI-MS m/z found for C_23_H_13_Cl_3_N_4_O_3_: 499.73 (M^+^); Anal. calcd for C_23_H_13_Cl_3_N_4_O_3_: C 55.28, H 2.62, Cl 21.28, N 11.21, O 9.60 found C 55.29, H 2.65, N 11.17, O 9.58.

#### Ethyl 2-((2-hydroxy 3-(2,5-dichlorophenyldiazenyl)naphthalen-1-yl)methyleneamino)-4,5,6,7-tetrahydrobenzo[*b*]thiophene-3-carboxylate (NS-6)

Maroon crystals; Yield: 68%; R_f_ = 0.57 (hexane/ethyl acetate 7:2); mp: 124–126 °C; IR (ATR, cm^−1^) ν_max_: 3568.00, 3094.03, 3040.81, 2974.81, 2911.20, 2366.67, 1700.69, 1606.35, 1475.69, 1371.47, 1297.35, 1196.50, 997.64, 870.26, 794.90; ^1^H NMR (300 MHz, CDCl_3_) δ: 14.83 (s, 1H), 9.29 (s, 1H), 8.11 (d, *J *= 11.2 Hz, 1H), 7.71–7.83 (m, 2H), 7.52–7.57 (m, 1H), 7.33–7.39 (m, 1H), 7.15–7.27 (m, 3H), 4.44 (q, *J *= 9.6 Hz, 2H), 2.74–2.82 (m, 4H), 1.44–1.84 (m, 4H), 1.43 (t, *J *= 9.6 Hz, 3H); ^13^C NMR (75 MHz, CDCl_3_) δ: 168.30, 166.54, 165.28, 163.52, 156.52, 156.21, 153.19, 138.89, 138.77, 135.86, 135.74, 133.82, 132.64, 132.31, 130.95, 130.78, 129.29, 128.99, 127.92, 127.73, 126.72, 123. 68, 122.44, 120.66, 119.42, 112.66, 110.63, 63.79, 26.34, 25.91, 25.56, 22.88, 22.54, 17.50, 14.48; APCI-MS m/z found for C_28_H_23_Cl_2_N_3_O_3_S: 551.08 (M^+^); Anal. calcd for C_28_H_23_Cl_2_N_3_O_3_S: C 60.87, H 4.20 Cl 12.83, N 7.61, O 8.69 S 5.80 found C 60.85, H 4.19 N 7.63, O 8.68.

#### 1-((2,5-Dichlorophenylimino)methyl)-3-((2,4-dimethylphenyl)diazenyl)naphthalen-2-ol (NS-7)

Orange color, Yield: 63%; R_f_ = 0.52 (hexane/ethyl acetate 3:2); mp: 136–138 °C; IR (ATR, cm^−1^) ν_max_: 3375.92, 3151.83, 3088.46, 2943.78, 2873.15, 1719.05, 1635.79, 1544.30, 1463.04, 1400.54, 130.27, 1245.55, 1177.83, 1131.88, 1074.45, 996.33, 940.63, 895.69, 754.43, 665.50; ^1^H NMR (400 MHz, DMSO-d_6_) δ: 15.53 (s, 1H), 9.83 (s, 1H), 8.64 (s, 1H), 8.34 (d, *J *= 4.2 Hz, 1H), 8.02 (d, *J *= 9.2 Hz, 1H), 7.85 (d, *J *= 8 Hz, 1H), 7.38–7.67 (m, 6 H), 7.11 (d, *J *= 9.2 Hz, 1H), 2.49 (s, 3H), 2.39 (s, 3H); ^13^C NMR (100 MHz, DMSO-d_6_) δ: 168.49, 159.07, 143.82, 137.79, 133.46, 133.39, 131.60, 129.50, 128.68, 127.66, 127.57, 126.43, 124.43, 121.53, 121.43, 120.33, 109.87, 25.69, 15.31; APCI-MS m/z found for C_28_H_23_Cl_2_N_3_O_3_S: 551.08 (M^+^); Anal. calcd for C_25_H_19_Cl_2_N_3_O: C 66.97, H 4.27 Cl 15.82, N 9.37, O 3.57 S 5.80 found C 60.85, H 4.19 N 7.63, O 8.68.

#### ((*E*)-Ethyl 2-((2-hydroxy 3-(2,5 dichlorophenyldiazenyl)naphthalen-1-yl)methyleneamino)-4-(2-thienyl) thiophene-3-carboxylate (NS-8)

Red Fluffy; Yield: 56%; mp: 150–152 °C; R_f_ = 0.64 (hexane/ethyl acetate 4:1); IR (ATR, cm^−1^) ν_max_: 3411.22, 3362.17, 3297.94, 3146.85, 3024.30, 2927.92, 2875.89, 1813.05, 1728.86, 1651.67, 1570.54, 1455.02, 1403.86, 1353.24, 1302.51, 1118.90, 1071.95, 963.19, 823.50, 751.85, 668.34, 623.73; ^1^H NMR (400 MHz, DMSO-d_6_) δ:14.58 (s, 1H), 9.45 (s, 1H), 8.44 (d, *J *= 8 Hz, 1H), 8.16 (s, 1H), 8.03 (d, *J *= 7.6 Hz, 1H), 7.89 (d, *J *= 2.4 Hz, 1H), 7.58–7.70 (m, 5H), 7.43 (t, *J *= 7.2 Hz, 1H), 7.19 (d, *J *= 9.2 Hz, 1H), 6.82 (d, *J *= 9.2 Hz, 1H), 4.32 (q, *J *= 7.2 Hz, 2H), 1.35 (t, *J *= 7.2 Hz, 3H); ^13^ C NMR (100 MHz, DMSO-d_6_) δ: 167.66, 161.40, 156.69, 154.69, 144.15, 141.26, 139.16, 137.19, 136.30, 133.36, 129.40, 128.36, 127.37, 123.91, 123.06, 121.55, 121.09, 120.74, 118.35, 116.22, 109.22, 63.14, 17.65; APCI-MS m/z found for C_28_H_19_Cl_2_N_3_O_3_S_2_: 579.08 (M^+^); Anal. calcd for C_28_H_19_Cl_2_N_3_O_3_S_2_: C 57.93, H 3.30, Cl 12.21, N 7.24, O 8.27, S 11.05 found C 57.95, H 3.29, N 7.23, O 8.28.

#### 1-((2-chloro-4-nitrophenylimino)methyl)-3-((2,4-dichlorophenyl)diazenyl)naphthalen-2-ol: (NS-9)

Orange color, Yield: 75%; mp: 145–148 °C; R_f_ = 0.56 (hexane/ethyl acetate 3:2); IR: 3397.56, 2888.01, 2706.56, 2624.92, 1674.85, 1561.71, 1499.61, 1332.79, 1291.62, 1123.56, 1071.35, 951.05, 888.01, 826.51, 742.29; ^1^H NMR (400 MHz, DMSO-d_6_) δ: 16.07 (s, 1H), 9.80 (s, 1H), 8.57 (d, *J *= 8.0 Hz, 1H), 8.48 (s, 1H), 8.34 (s, 1H), 8.16 (d, *J *= 2.4 Hz, 1H), 7.99–8.01 (m, 1H), 7.77 (d, *J *= 8.0 Hz, 1H), 7.63–7.71 (m, 1H), 7.52 (t, *J *= 7.6 Hz, 1H), 7.37–7.40 (m, 1H), 7.10 (s, 1H), 6.84 (d, *J *= 10 Hz, 1H); ^13^C NMR (100 MHz, DMSO-d_6_): 166.71, 163.56, 157.72, 153.25, 151.87, 145.69, 143.29, 141.45, 138.56, 136.34, 132.02, 130.29, 129.76, 127.28, 126.25, 125.59, 125.43, 124.31, 122.77, 116.72, 109.78; APCI-MS m/z found for C_23_H_13_Cl_3_N_4_O_3_: 499 (M^+^); Anal. calcd for C_23_H_13_Cl_3_N_4_O_3_: C 55.28., H 2.62, Cl 21.28, N 11.21, O 9.60 found C 55.31, H 2.59, N 11.17, O 9.57.

#### ((*E*)-Ethyl 2-((2-hydroxy 3-(2,5 dichlorophenyldiazenyl)naphthalen-1-yl)methyleneamino)-4-(2, 4 dihydroxyphenylthienyl) thiophene-3-carboxylate (NS-10)

Maroon color, Yield: 63%; R_f_ = 0.74 (hexane/ethyl acetate 3:2); mp: 130–133 °C; IR (ATR, cm^−1^) ν_max_: 3426.91, 3310.91, 3171.12, 3069.50, 1828.70, 1747.69, 1680.17, 1563.58, 1515.21, 1454.32, 1379.35, 1283.47, 1116.01, 989.49, 861.16, 747.55, 688.55, 633.85; ^1^H NMR (400 MHz, DMSO-d_6_) δ: 16.08 (s, 1H), 12.11–12.34 (m, 2H), 9.75 (s, 1H), 8.58 (d, *J *= 8 Hz, 1H), 8.22 (d, *J *= 7.6 Hz, 1H), 8.16 (d, *J *= 2.4 Hz, 1H), 8.18 (d, *J *= 2.4 Hz, 1H), 7.79 (d, *J *= 7.6 Hz, 1H), 7.53–7.77 (m, 2H), 7.50–7.52 (m, 2H), 7.16–7.39 (m, 2H), 6.82 (d, *J *= 7.6 Hz, 1H), 4.32 (q, *J *= 7.2 Hz, 2H), 1.35 (t, *J *= 7.2 Hz, 3H); ^13^C NMR (100 MHz, DMSO-d_6_) δ: 168.56, 163.96, 159.46. 157.20, 155.25, 150.88, 148.27, 144.62, 141.54, 133.71, 131.56, 131.11, 130.47, 129.45, 129.28, 127.80, 127.65, 127.45, 124.98, 116.03, 114.27, 108.81, 102.09, 61.06, 14.43; APCI-MS m/z found for C_30_H_21_Cl_2_N_3_O_5_S: 605 (M^+^); Anal. calcd for C_30_H_21_Cl_2_N_3_O_5_S: C 59.41, H 3.49; Cl 11.69, N 6.93, O 13.19, S 5.29 found C.59.43, H 3.48, N 6.94, O 13.178.

#### (*E*)-3-((2,5-dichlorophenyl)diazenyl)-1-((furan-2-ylmethylimino)methyl)naphthalen-2-ol (NS-11)

Maroon color, Yield: 64%; R_f_ = 0.44 (hexane/ethyl acetate 3:2); mp: 68–70 °C; IR (ATR, cm^−1^) ν_max_: 3367.69, 3254.68, 3135.41, 2993.51, 2888.91, 2353.45, 1664.24, 1591.44, 1484.94, 1386.94, 1322.25, 1202.01, 1082.30, 1003.24, 916.27, 813.44, 731.84, 663.67; ^1^HNMR (400 MHz, DMSO-d_6_) δ: 14.02 (s, 1H), 8.94 (d, *J *= 8.4 Hz, 1H), 8.51 (s, 1H), 8.14 (d, *J *= 8.4 Hz, 1H), 7.89 (d, *J *= 8.0 Hz, 1H), 7.62 (t, *J *= 8.0 Hz, 1H), 7.43 (t, *J *= 8.0 Hz, 1H), 7.38–7.40 (m, 1H), 7.43 (t, *J *= 8.0 Hz, 1H), 7.38–7.40 (m, 1H), 7.23 (d, *J *= 9.2 Hz, 1H), 6.53 (s, 1H), 6.45 (d, *J *= 1.6 Hz, 1H), 4.76 (s, 2H); ^13^C NMR (100 MHz, DMSO-d_6_): 166.14, 160.23, 155.34, 145.27, 142.29, 141.27, 134.17, 133.13, 130.80, 129.47, 128.95, 128.46, 126.98, 126.10, 125.83, 122.28, 116.75, 112.75, 109.53, 54.51; APCI-MS m/z found for C_22_H_15_Cl_2_N_3_O_2_: 423 (M^+^); Anal. calcd for C_22_H_15_Cl_2_N_3_O_2_: C 62.28., H 3.56; Cl 16.71, N 9.90, O 7.54 found C 62.26, H 3.52 N 9.89, O 7.56.

#### (*E*)-3-((2,5-dichlorophenyl)diazenyl)-1-((4-methylpyridin-2-ylimino) methyl)naphthalen-2-ol) (NS-12)

Red color; Yield: 67%; R_f_ = 0.52 (hexane/ethyl acetate 3:2); mp: 70–72 °C; IR (ATR, cm^−1^) ν_max_: 3429.20, 3310.25, 3063.91, 1681.87, 1611.58, 1546.17, 1463.44, 1328.96, 1255.15, 1074.37, 876.25, 802.90, 729.57; ^1^H NMR (400 MHz, DMSO-d_6_): 16.08 (s, 1H), 9.82 (s, 1H), 8.93 (d, *J *= 8.4 Hz, 1H), 8.22 (d, *J *= 7.6 Hz, 1H), 7.79 (d, *J *= 7.6 Hz, 1H), 7.53–7.77 (m, 5H), 7.50–7.52 (m, 2H), 7.36–7.39 (m, 2H), 6.82 (d, *J *= 2.4 Hz, 1H), 2.28 (s, 3H); ^13^C NMR (100 MHz, DMSO-d_6_): 166.34, 164.15, 160.82, 157.92, 154.92, 143.89, 141.42, 139.12, 135.61, 134.27, 132.64, 131.29, 130.11, 130.00, 128.10, 124.62, 122.72, 121.02, 120.63, 115.87, 109.86, 27.06; APCI-MS m/z found for C_23_H_16_Cl2N_4_O: 434 (M^+^); Anal. calcd for C_23_H_16_Cl_2_N_4_O: C 63.46, H 3.70, Cl 16.29, N 12.87, O 3.68 found C 63.48, H 3.72. N 12.89, O 3.69.

#### (*E*)-4-((4-((4-chloro-2-nitrophenylimino)methyl)-3-hydroxynaphthalen-2-yl)diazenyl) benzoic acid (NS-13)

Orange color; Yield: 64%; mp: 58–60 °C; R_f_ = 0.52 (hexane/ethyl acetate 3:2); ^1^HNMR (400 MHz, DMSO-d_6_) δ:14.57 (s, 1H), 12.99 (s, 1H), 9.71 (s, 1H), 8.57 (d, *J *= 8.4 Hz, 1H), 8.48 (d, *J *= 8.4 Hz, 1H), 8.24 (d, *J *= 2.4 Hz, 1H), 8.12 (d, *J *= 2.4 Hz, 1H), 8.05 (t, *J *= 8.8 Hz, 1H), 7.85–7.98 (m, 2H), 7.75 (d, *J *= 7.6 Hz, 1H 1H), 7.43–7.63 (m, 2H), 7.10 (d, *J *= 9.2 Hz, 1H), 6.80 (d, *J *= 9.6 Hz, 1H); ^13^C NMR (100 MHz, DMSO-d_6_) δ: 170.26, 164.25, 162.19, 152.36, 142.14, 138.56, 138.23, 134.79, 133.16, 142.14, 138.56, 138.23, 134.79, 133.16, 132.0, 131.56, 129.29, 128.06, 127.90, 126.49, 125.41, 124.96, 118.89, 114.02; APCI-MS m/z found for C_24_H_15_ClN_4_O_5_: 474.8 (M^+^); Anal. calcd for C_24_H_15_ClN_4_O: C 60.70, H 3.18, Cl 7.47, N 11.80, O 16.85 found C 60.72, H 3.19, N 11.83, O 16.81.

#### (*E*)-Ethyl 2-((2-hydroxy 3-(2-nitro 4-chloro phenyldiazenyl)naphthalen-1-yl)methyleneamino)-4,5,6,7-tetrahydrobenzo[*b*]thiophene-3-carboxylate (NS-14)

Red crystals, Yield: 72%; R_f_ = 0.57 (hexane/ethyl acetate 5:2); mp: 128–130 °C; IR (ATR, cm^−1^) ν_max_: 3417.23, 2880.53, 2518.88, 1721.43, 1630.61, 1461.73, 1354.60, 1245.60, 1115.48, 1074.41, 930.64, 820.99, 662.00; ^1^H NMR: (300 MHz, CDCl_3_) δ: 14.83 (s, 1H), 9.30 (s, 1H), 8.34 (s, 1H), 8.27 (s, 1H), 8.11 (d, *J *= 9.2 Hz, 1H), 7.73–7.82 (m, 1H), 7.54 (s, 1H), 7.26–7.35 (m, 2H), 7.17 (d, *J *= 11.6 Hz,, 1H), 4.44 (d, *J *= 9.2 Hz, 2H), 2.62–2.82 (m, 4H), 1.61–1.84 (m, 4H), 1.42 (t, *J *= 9.2 Hz, 3H); ^13^C NMR (75 MHz, CDCl_3_) δ: 165.29, 163.55, 153.66, 153.16, 135.94, 135.79, 132.68, 130.81, 129.34, 127.97, 127.79, 123.73, 123.29, 122.68, 118.43, 116.67, 109.55, 60.77, 26.34, 25.58, 22.91, 22.55, 14.46; APCI-MS m/z found for C_28_H_23_ClN_4_O_5_S: 563 (M^+^); Anal. calcd for C_28_H_23_ClN_4_O_5_S: C 59.73, H 4.12 Cl 6.30, N 9.95, O 14.21, S 5.70 found C 59.79, H 4.14, N 9.97, O 14.23.

#### 3-((4-hloro-2-nitrophenyl)diazenyl)-1-((4-methylpyridin-2-ylimino)methyl)naphthalen-2-ol (NS-15)

Maroon crystals, Yield: 61%; R_f_ = 0.49 (hexane/ethyl acetate 3:2); mp: 65–70 °C; IR (ATR, cm^−1^) ν_max_: 3385.46, 3304.04, 3236.92, 3084.45, 2923.61, 2859.42, 1724.04, 1629.08, 1462.98, 1397.92, 1314.26, 1251.36, 1171.02, 1079.03, 972.11, 915.89, 822.63, 744.24, 624.86; ^1^H NMR (300 MHz, CDCl_3_) δ: 13.21 (s, 1H), 9.85 (s, 1H), 8.66 (d, *J *= 9.6 Hz, 1H), 8.54 (d, *J *= 10 Hz, 1H), 8.34 (d, *J *= 9.6 Hz, 1H), 7.98 (t, *J *= 5.6 Hz, 1H), 7.79 (d, *J *= 10 Hz, 1H), 7.59–7.64 (m, 2H), 7.41–7.46 (m, 2H), 7.12–7.26 (m, 2H), 2.09 (s, 3H); ^13^C NMR (75 MHz, CDCl_3_) δ: 166.27, 164.50, 160.25, 158.36, 147.31, 145.18, 142.21, 139.32, 135.12, 132.65, 129.48, 129.12, 127.79, 124.51, 119.17, 118.55 116.38, 109.45; APCI-MS m/z found for C_23_H_16_ClN_5_O_3_: 445 (M^+^); Anal. calcd for C_23_H_16_ClN_5_O_3_: C 61.96, H 3.62 Cl 7.95, N 15.71, O 10.77 found C 61.93, H 3.631 N 15.68, O 10.76.

#### (*E*)-Ethyl 2-((2-hydroxy 3-(2-chloro 4-nitro phenyldiazenyl)naphthalen-1-yl)methyleneamino)-4,5,6,7-tetrahydrobenzo[*b*]thiophene-3-carboxylate (NS-16)

Maroon shiny crystals, Yield: 73%; R_f_ = 0.51 (hexane/ethyl acetate 3:2); mp: 138–140 °C; IR (ATR, cm^−1^) ν_max_; 3457.37, 3327.03, 3126.54, 3029.09, 2340.22, 1665.15, 1573.53, 1493.45, 1431.53, 1325.02, 1255.94, 1187.72, 1127.49, 995.00, 824.71, 746.41, 653.87; ^1^H NMR (300 MHz, CDCl_3_) δ: 14.83 (s, 1H), 9.30 (s, 1H), 8.32 (s, 1H), 8.10 (d, *J *= 11.2 Hz, 1H), 7.81 (d, *J *= 11.2 Hz, 1H), 7.74 (d, *J *= 11.2 Hz, 1H), 7.54 (t, *J *= 9.6 Hz, 1H), 7.36 (t, *J *= 9.6 Hz, 1H), 7.16–7.26 (m, 2H), 4.44 (q, *J *= 9.6 Hz, 2H), 2.74–2.82 (m, 4H), 1.59–1.84 (m, 4H), 1.43 (t, *J *= 9.6 Hz, 3H); ^13^C NMR (75 MHz, CDCl_3_) δ: 165.28, 163.55, 153.66, 153.16, 135.94, 135.79, 132.68, 130.81,129.34, 127.97, 127.78, 123.73, 123.29, 122.68, 118.43, 116.67, 109.55, 60.79, 26.34, 25.58, 22.91, 22.55, 14.44; APCI-MS m/z found for C_18_H_23_ClN_4_O_5_S: 563 (M^+^); Anal. calcd for C_18_H_23_ClN_4_O_5_S: C 59.73, H 4.12 Cl 6.30, N 9.95, O 14.21, S 5.70 found C 59.72, H 4.11, N 6.26, O 14.23.

#### 4-((2-Hydroxy 3(-(2-nitro 4-chloro phenyldiazenyl)naphthalen-1-yl)methyleneamino) butanoic acid (NS-21)

Brown color; Yield: 70%; R_f_ = 0.41 (hexane/ethyl acetate 3:2); mp: 140–145 °C IR (ATR, cm^−1^) ν_max_: 3433.92, 3352.25, 3294.64, 3157.07, 3078.15, 3028.37, 2879.17, 1673.84, 1621.20 1595.60, 1430.35, 1360.25, 1281.07, 1223.76, 1119.36, 1071.34, 932.88, 844.53, 766.60, 693.04, 624.04; ^1^H NMR (400 MHz, DMSO): 14.12 (s, 1H), 12.17 (s, 1H) 9.10 (s, 1H), 8.43 (d, *J *= 8.0 Hz, 1H), 8.31 (s, 1H), 8.06 (d, *J *= 8.0 Hz, 1H), 7.72 (d, *J *= 9.6 Hz, 1H), 7.63 (d, *J *= 7.6 Hz, 1H), 7.40–7.43 (m, 1H), 7.17–7.20 (m, 1H), 6.72 (d, *J *= 9.2 Hz, 1H), 3.66 (t, *J *= 6.8 Hz, 2H), 2.32 (t, *J *= 7.2 Hz, 2H), 1.90 (t, *J *= 6.8 Hz, 2H); ^13^C NMR (75 MHz, CDCl_3_) δ: 173.26, 165.93, 161.91, 153.65, 142.23, 139.13, 132.87, 129.48, 129.12, 127.79, 124.51, 119.17, 118.59, 116.28, 109.36, 59.23, 33.17, 26.14; APCI-MS m/z found for C_21_H_17_ClN_4_O_5_: 440 (M^+^); Anal. calcd for C_21_H_17_ClN_4_O_5_; C 57.22, H 3.89 Cl 8.04, N 12.71, O 18.15 found C 57.25, H 3.87 N 12.67, O 18.13.

#### 4-((2-hydroxy 3-(2,5 dichlorophenyldiazenyl)naphthalen-1-yl)methyleneamino)butanoic acid (NS-22)

Orange color; Yield: 74%; R_f_ = 0.48 (hexane/ethyl acetate 5:2); mp: 140–142 °C; IR (ATR, cm^−1^) ν_max_: 3554.29, 3482.86, 3275.53, 3163.02, 3043.78, 2923.95, 2785.48, 1683.59, 1626.14, 1560.99, 1490.84, 1416.86, 1360.38, 1206.58, 1098.88, 983.42, 867.13, 800.46, 728.61, 662.08; ^1^H NMR (300 MHz, CDCl_3_) δ: 14.81 (s, 1H), 12.51 (s, 1H), 9.21 (s, 1H), 8.51 (d, *J *= 8.0 Hz, 1H), 8.25 (s, 1H), 8.12 (d, *J *= 8 Hz, 1H), 7.77 (d, *J *= 9.6 Hz, 1H), 7.46–7.63 (m, 2H), 7.13–7.25 (m, 1H), 6.79 (d, *J *= 9.2 Hz, 1H), 3.62 (t, *J *= 7.6 Hz, 2H), 2.52 (t, *J *= 7.6 Hz, 2H), 1.95 (t, *J *= 7.6 Hz, 2H); ^13^C NMR (75 MHz, CDCl_3_) δ: 173.26, 165.93, 161.91, 153.65, 142.23, 139.13, 132.87, 129.48, 129.12, 127.79, 124.51, 119.17, 118.59, 116.28, 109.36, 59.23, 33.17, 26.14; APCI-MS m/z found for C_21_H_17_Cl_2_N_3_O_3_: 430 (M^+^); Anal. calcd for C_21_H_17_Cl_2_N_3_O_3_; C 58.62, H 3.98 Cl 16.48, N 9.77, O 11.16 found C 58.65, H 3.99, N 9.79, O 11.14.

#### 4-((2-hydroxy 3-(4-carboxydiazenyl)naphthalen-1-yl)methyleneamino)butanoic acid (NS-23)

Orange color; Yield: 77%; R_f_ = 0.49 (hexane/ethyl acetate 3:2); mp: 134–136 °C; IR (ATR, cm^−1^) ν_max_: 3346.74, 3305.52, 2847.68, 2770.40, 2265.75, 1721.84, 1633.06, 1461.09, 1411.46, 1310.26, 1252.90, 1173.70, 1096.01, 974.77, 827.65, 752.59, 666.23.14, 1560.99, 1490.84, 1416.86, 1360.38, 1206.58, 1098.88, 983.42, 867.13, 800.46, 728.61, 662.08; ^1^H NMR (300 MHz, CDCl_3_) δ: 14.81 (s, 1H), 11.89–12.01 (m, 2H), 8.75 (s, 1H), 7.86 (d, *J *= 8 Hz, 1H), 7.59–7.68 (m, 3H), 7.22–7.47 (m, 4H), 6.92 (d, *J *= 12.2 Hz, 1H), 3.72 (t, *J *= 8.0 Hz, 2H), 2.54 (t, *J *= 8.0 Hz, 2H), 2.13 (t, *J *= 8.0 Hz, 2H); ^13^C NMR (75 MHz, CDCl_3_) δ: 177.26, 170.25, 164.27, 163.52, 153.56, 151.27, 135.89, 135.64, 132.56, 130.70, 129.37, 127.64, 123.76, 123.14, 120.56, 116.42, 58.77, 35.33, 26.99; APCI-MS m/z found for C_22_H_19_N_3_O_5_: 405.08 (M^+^); Anal. calcd for C_22_H_19_N_3_O_5_: C 65.18, H 4.72, N 10.37, O 19.73 found C 65.21, H 4.74, N 10.34, O 19.76.

### Antimicrobial activity

#### Determination of MIC

The MIC values of synthesized Schiff bases were determined by tube dilution method by the reported procedure [38]. The fluconazole (antifungal) and cefotaxime (antibacterial) were used as standard drugs. The test compounds and standard drugs were dissolved in DMSO to get stock solutions of the concentration of 1000 μg/ml. The test and standard compounds were serially diluted to different concentrations (500, 250, 125, 62.5, 31.25, 15.62, 7.81, 3.90, 1.95 μg/ml) in nutrient broth (for bacterial strains) and sabouraud dextrose broth (for fungal strains). The 100 μl of microbial inoculum was added to each concentration of the test and standard compounds to give final inoculum size of 5 × 10^5^ colony forming units (CFU) ml^−1^ under sterile conditions. The different tubes with various concentration of the test and standard compounds and microbial strains were incubated for the specified time (for bacterial cultures-24 h at 37 ± 2 °C; fungal cultures-7 days at 25 ± 2 °C.

#### Determination of MBC/MFC

After MIC evaluation, the synthesised derivatives **NS1**–**NS23**, were further assessed for MBC and MFC values. To the sterilized petri plates, added 100 µl of culture from each test tube showing no visible growth in MIC test tubes aseptically. The 10–15 ml of nutrient agar and Sabouraud dextrose agar was added to the petri plates for bacterial and fungal samples respectively with gentle shaking of plates in order to mix the culture throughout the media. Allowed the media to solidify. The petri plates were then incubated for the specified time and temperature as mentioned previously for bacterial and fungal cultures respectively. The plates were then analysed visually for growth. The MBC and MFC were stated as the minimum concentration of the compounds in aliquots showing no visual growth after incubation.

### Cytotoxicity study

#### Cell culture

HT-29 cell line was initially procured from the National Centre for Cell Sciences (NCCS), Pune, India, and maintained in DMEM (Dulbecco’s Modified Eagle Medium). The cell line was cultured in 25 cm^2^ tissue culture flask with DMEM supplemented with 10% FBS (Fetal bovine serum), l-glutamine, sodium bicarbonate and an antibiotic solution containing: penicillin (100 U/ml), streptomycin (100 μg/ml). The cultured cell line was kept at 37 °C in a humidified 5% CO_2_ incubator (VWR, USA).

#### MTT cell proliferation assay

The compounds found to have good antimicrobial potential were then screened for their cytotoxicity using MTT assay [39, 40]. Aliquot (200 μl) suspension of cells was seeded in a 96-well plate at cell density of 2 × 10^4^ cells per well. The cells were incubated in a CO_2_ incubator for 24 h. Afterward, test compounds were added in the desired concentrations (5, 10, 15, 25, 50, 100 µg/ml) to the wells. Simultaneously, the culture medium without cells was used as medium control to neglect the interference from other reducing components such as cholesterol, ascorbic acid, etc, present in the medium. The culture medium without test compounds was used as a negative control. The plates were incubated for 24 h at 37 °C in a 5% CO_2_ atmosphere. After incubation, plates were removed and decanted off the spent medium followed by addition of MTT reagent to a final concentration of 0.5 mg/ml of total volume. The plates were again incubated for 3 h. The MTT reagent was removed followed by addition of 100 μl of solubilization solvent DMSO with stirring in a gyratory shaker. The absorbance was read on an ELISA reader at 630 nm. The IC_50_ value was calculated using the linear regression equation i.e. Y = Mx + C. Here, Y = 50, M and C values were derived from the viability graph. The assay was performed in duplicate.

#### Morphological study

The HT-29 cells were exposed to the indicated concentrations of the standard and test compounds and morphological changes were monitored after 24 h. The photographs were taken with an inverted phase microscope (Biolink).

#### Apoptosis study by flow cytometer

The induction of apoptosis by test compounds in HT-29 cells was confirmed by AV/PI staining assay using flow cytometer [41]. The cells were cultured in a 6-well plate at a density of 3 × 10^5^ cells/2 ml and incubated at 37 °C for 24 h in a CO_2_ incubator. The spent medium was aspirated and the cells were treated with the IC_50_ concentration of the test compounds (**NS-2** and **NS-21**) and standard, in 2 ml of culture medium and again incubated for 24 h. After incubation, the medium was removed from all the wells and cells were given phosphate buffer saline (PBS) wash. Afterward, 200 μl of trypsin–EDTA solution was added to the cells followed by incubation at 37 °C for 3–4 min. The 2 ml of culture medium was added to the cells and harvested directly into 12 × 75 mm polystyrene tubes. The tubes were centrifuged for 5 min at 300×*g* at 25 °C. The supernatant was decanted carefully. The cells were washed twice with PBS. Decant the PBS completely. 5 μl of FITC Annexin-V was added. The cells were vortexed gently and incubated for 15 min at room temperature (25 °C) in the dark. 5 μl of PI and 400 μl of 1X Binding buffer was added to each tube and vortexed gently. The cells were analyzed immediately after addition of PI by flow cytometry.

#### Cell cycle analysis

The most frequently used dye for DNA content/cell cycle analysis is PI. It can be used to stain whole cells or isolated nuclei [42]. The cells (1 × 10^5^) were seeded in 24 well plates and treated with the IC_50_ concentration of the test compounds for 24 h. At the end time, the cells were detached by trypsin–EDTA solution at 37 °C for 5 min. Next, the trypsin activity was stopped with adding 10% FBS–RPMI 1640 medium. Both adherent and detached cells were collected, washed in cold PBS twice, fixed by ice-cold ethanol (70% w/w) and then incubated in PBS containing 0.1%, Triton X-100, 0.1% sodium citrate, RNase A (50 μg/ml; Fermentas), and PI (50 μg/ml; Sigma) at 4 °C for 30 min. The percent of calculated cells in the sub-G1, G0/G1, S, and G2/M phases were analyzed by flow cytometry (BD FACSCalibur flow cytometer, USA).

## Conclusion

In search of novel dual-action drugs having the potential for colon cancers and microbial infections both, a series of novel naphthol diazenyl scaffold containing Schiff bases was efficiently synthesized and characterized by various spectroscopic techniques. During preliminary evaluation for antimicrobial potential, the derivatives **NS-2** and **NS-8** were found most active against bacterial strains *E. coli*, *S. enterica* with very low MIC and MBC values while **NS-21** and **NS-23** were found most active against fungal strain *A. fumigatus*. The derivatives showing good antimicrobial properties were further screened for their cytotoxic potential against human colorectal carcinoma cell line (HT-29). The test derivatives **NS-2** and **NS-21** exhibited significant cytotoxicity against HT-29 cell line with IC_50_ values from 4.8 µg/ml and 19.2 µg/ml respectively and further selected for evaluation of apoptosis-inducing potential in HT-29 cells. In conclusion, both **NS-2** and **NS-21** have induced apoptosis in HT-29 cell line particularly **NS-2** with more than 90% of the cells in the apoptotic phase after 24 h treatment as compared to 68% in case of standard drug doxorubicin. Both **NS-2** and **NS-21** have arrested the cells in S and G2/M phases of the cell cycle. On the basis of the above results, it is clear that these derivatives can have potential in therapeutics including treatment for both cancer and associated microbial infections simultaneously.

## Additional file

**Additional file 1.** ^1^H and ^13^C NMR data of most active compounds has been provided.

## Abbreviations

MIC: minimum inhibitory concentration
MBC: minimum bactericidal concentration
MFC: minimum fungicidal concentration
MTT: 3-(4,5-dimethylthiazol-2-yl)-2,5-diphenyltetrazolium bromide
*E. coli*: *Escherichia coli*
*S. enterica*: *Salmonella enterica*
*B. subtilis*: *Bacillus subtilis*
*S. aureus*: *Staphylococcus aureus*
*A. niger*: *Aspergillus niger*
*A. fumigatus*: *Aspergillus fumigatus*
AV: Annexin-V
PI: propidium iodide
PS: phosphatidylserine
DMEM: Dulbecco’s Modified Eagle Medium
PBS: phosphate buffer saline
FBS: fetal bovine serum
