# Supplementary material for: Synthesis and evaluation of novel naphthol diazenyl scaffold based Schiff bases as potential antimicrobial and cytotoxic agents against human colorectal carcinoma cell line (HT-29)
Source: BMC Chem. 2019 Apr 2;13(1):49. doi: 10.1186/s13065-019-0558-y (PMC6661811; doi:10.1186/s13065-019-0558-y)

**Synthesis and evaluation of novel naphthol diazenyl scaffold based Schiff bases as potential antimicrobial and cytotoxic agents against human colorectal carcinoma cell line (HT-29)**

Harmeet Kaur^a^, Jasbir Singh^b^, Balasubramanian Narasimhan^a^*

The ^1^H and ^13^C data of some selected compounds have been provided below


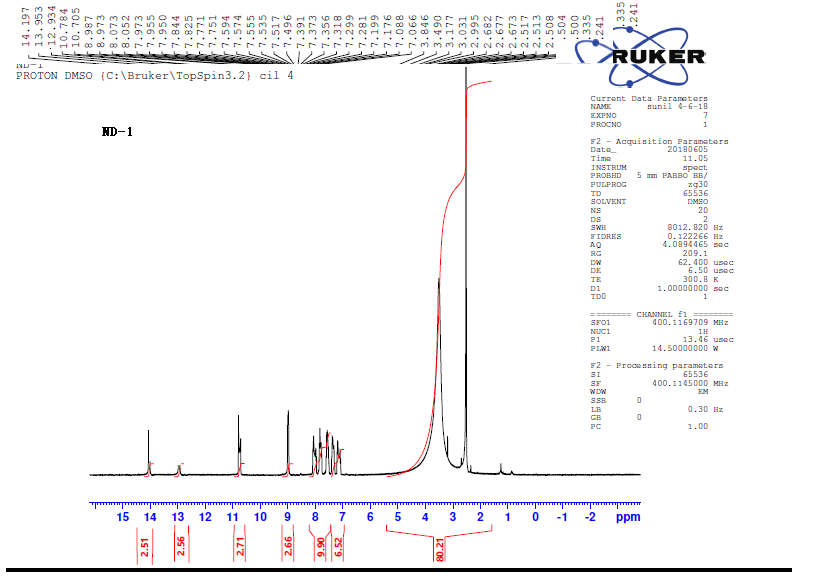


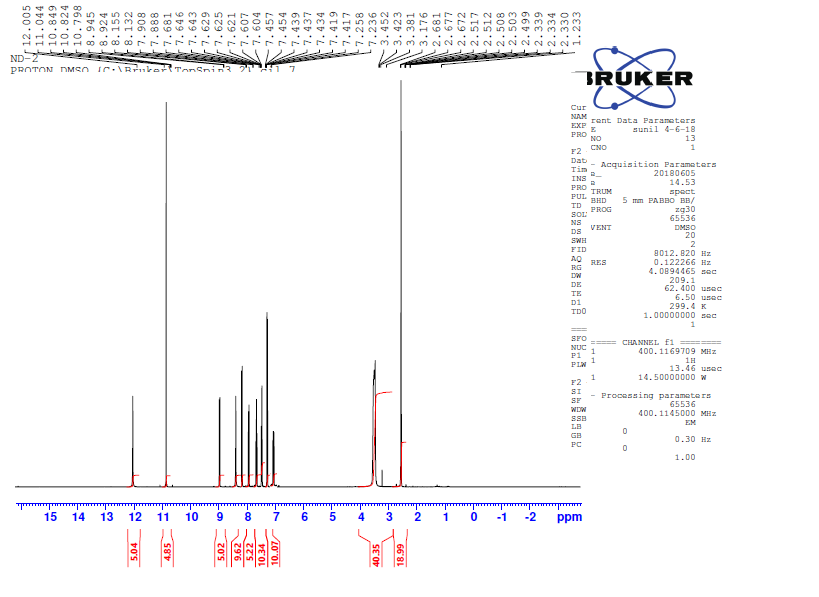


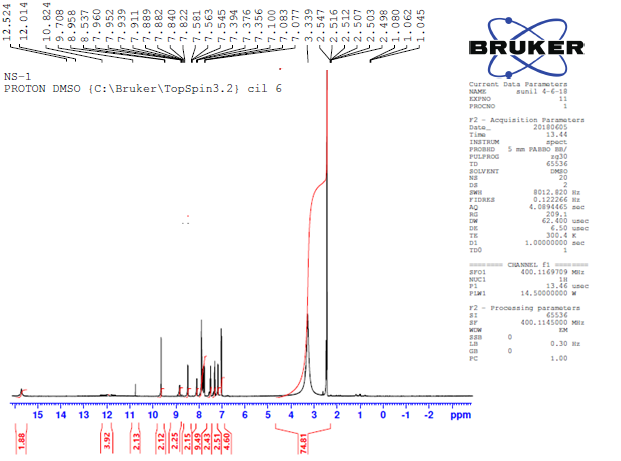


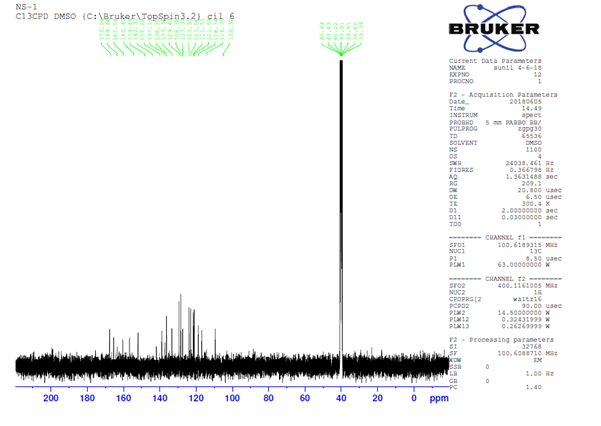


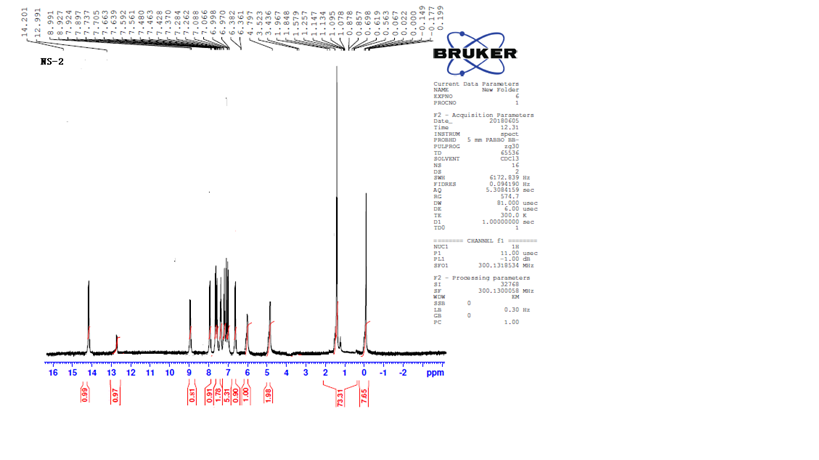

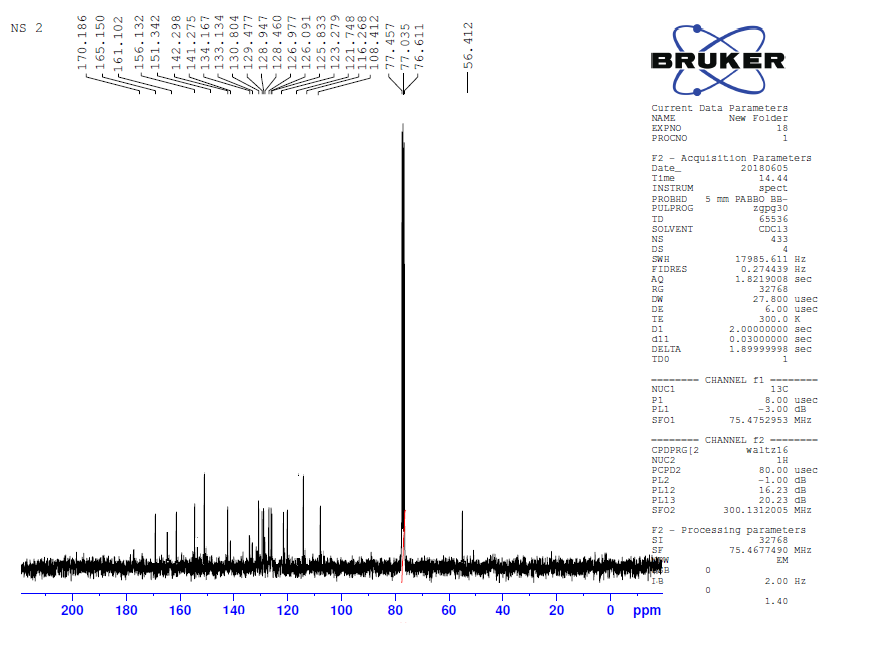


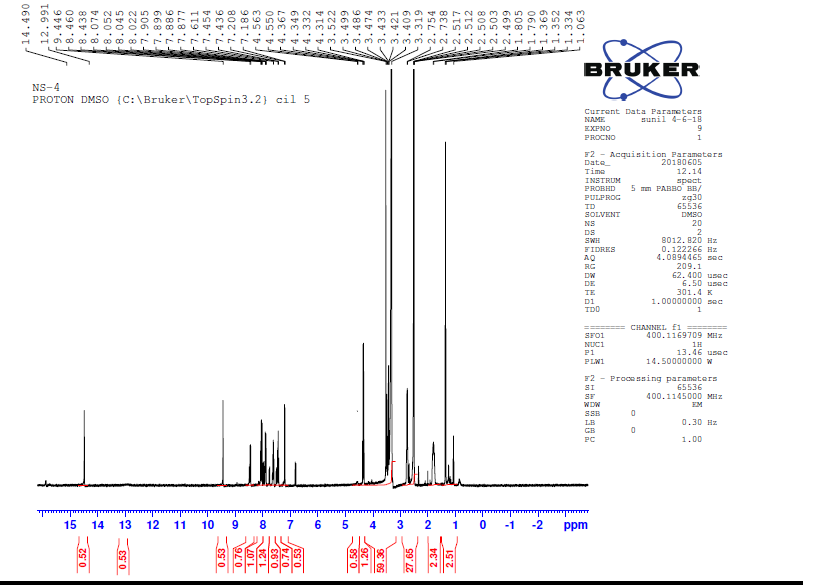


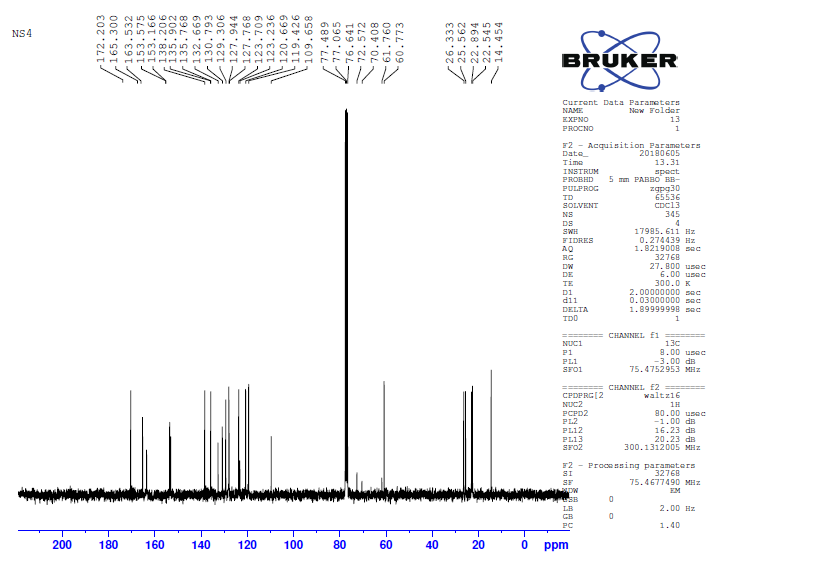

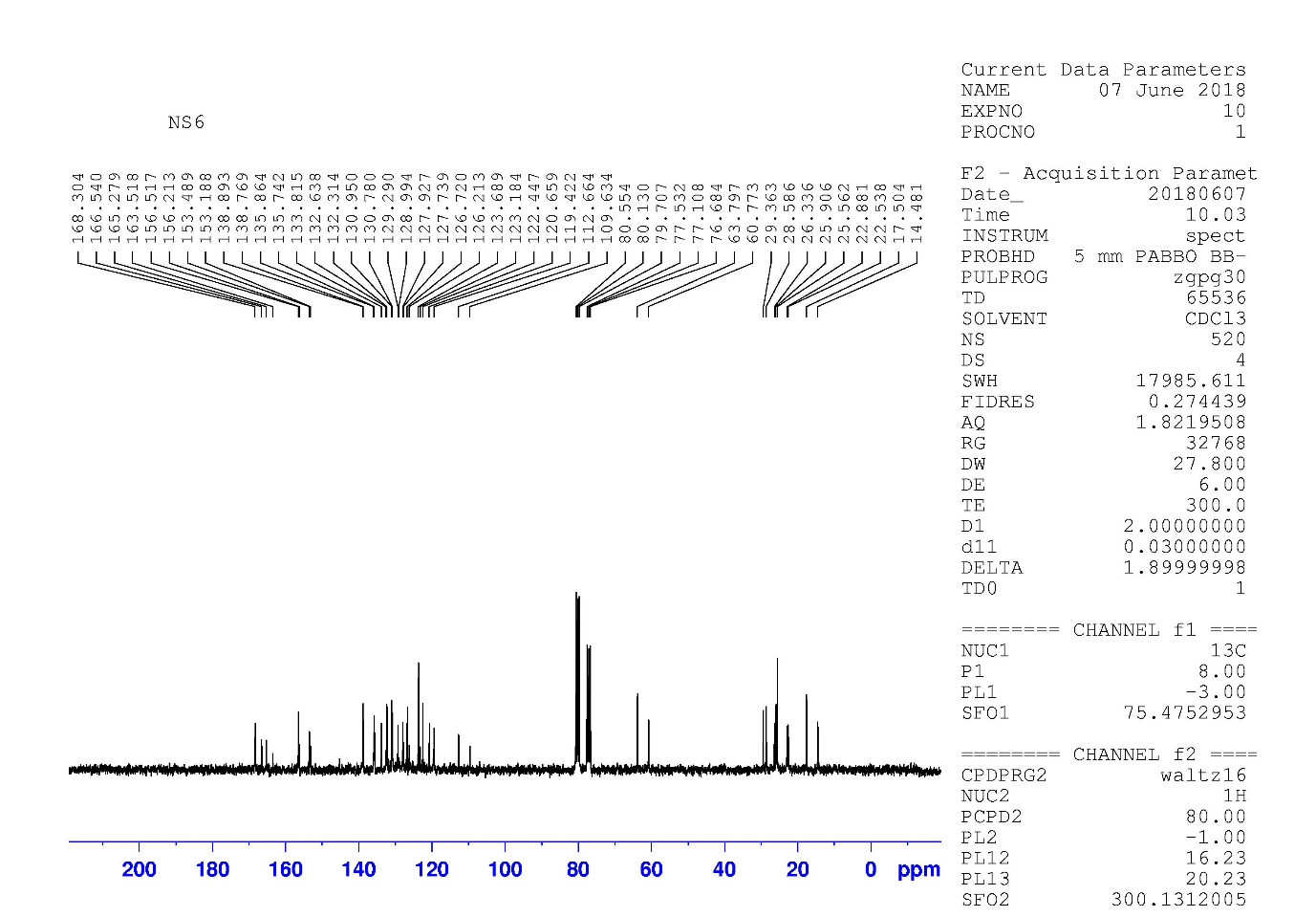


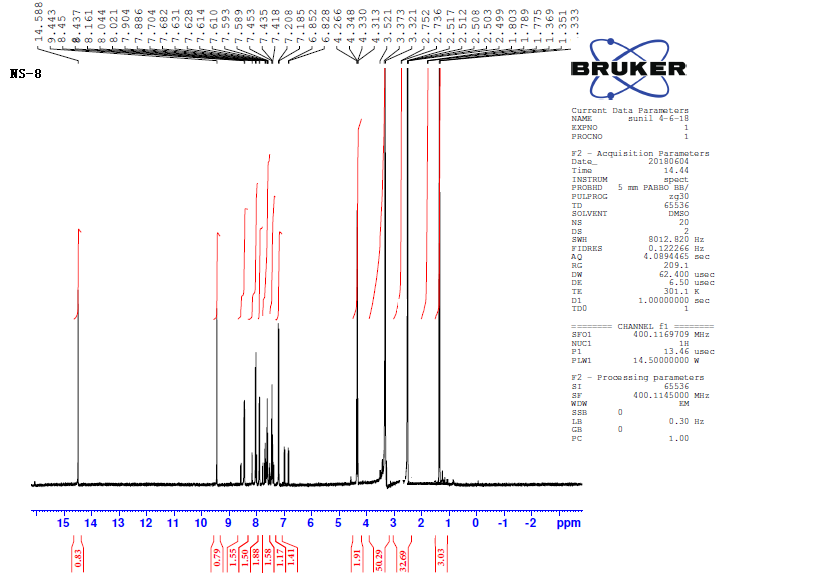


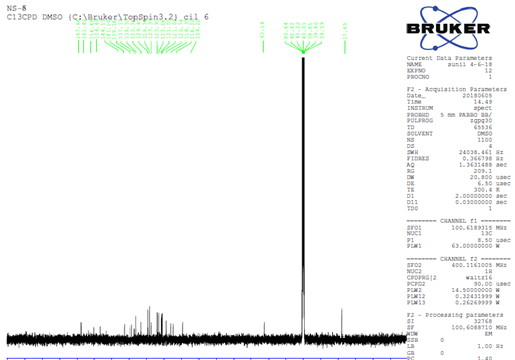


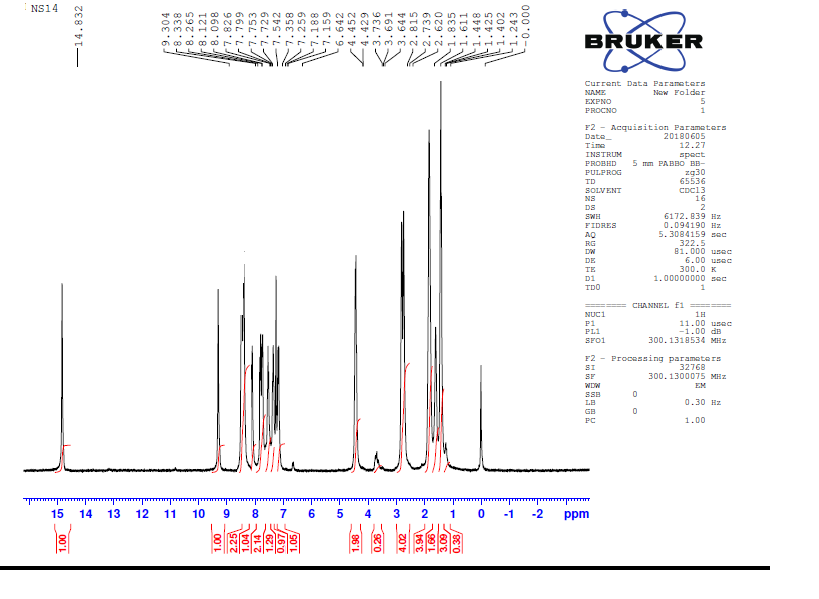


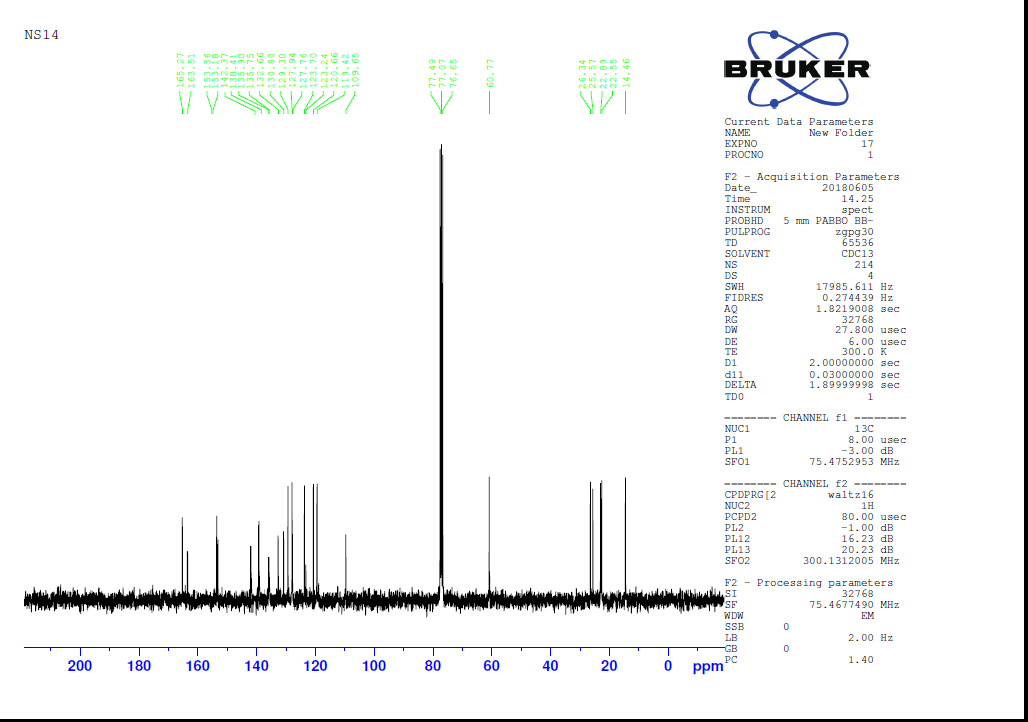


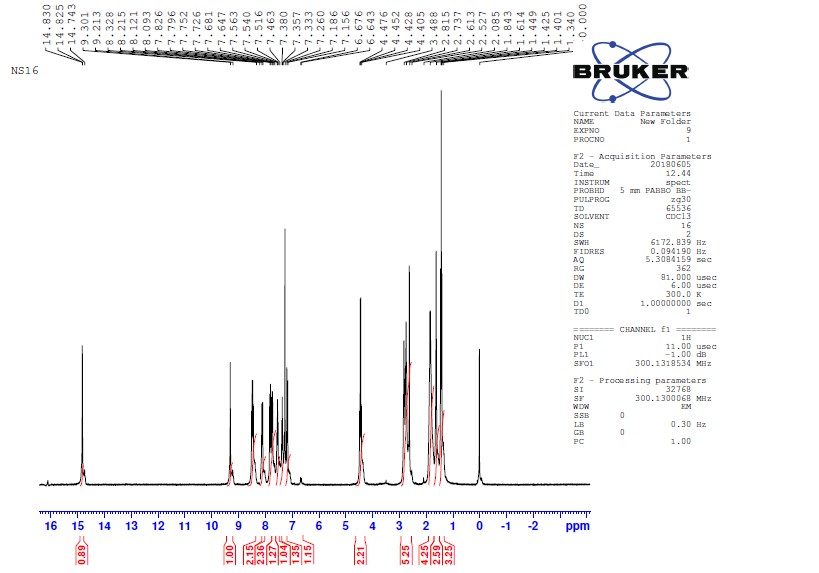


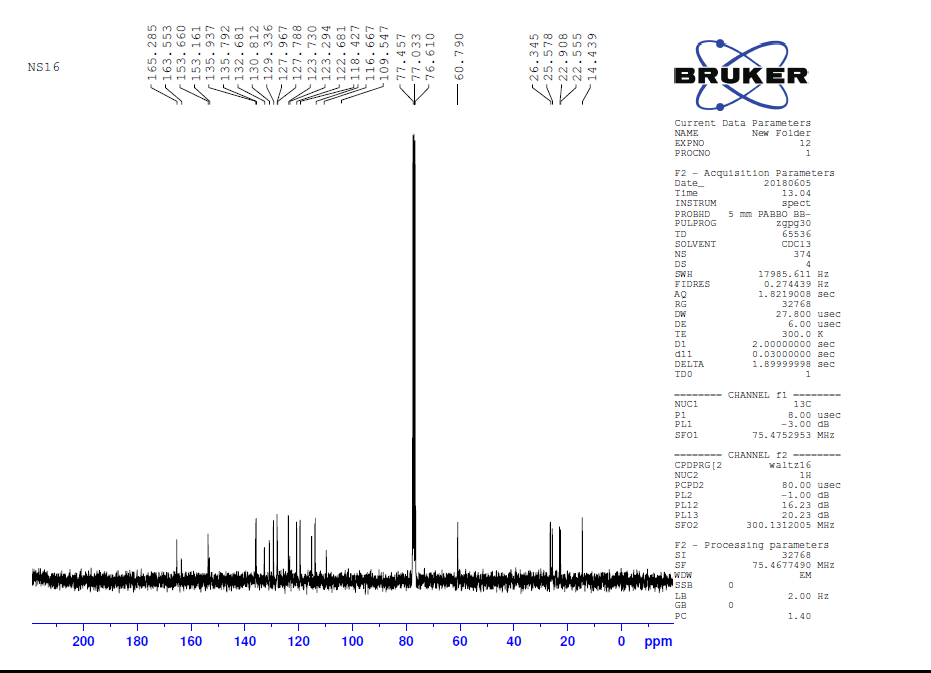


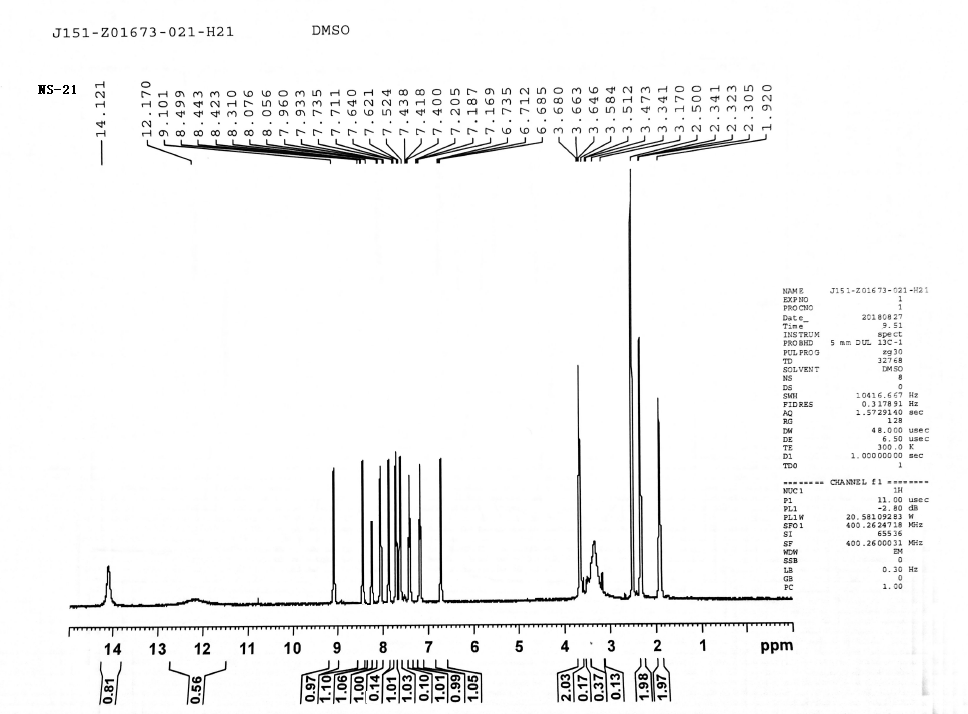


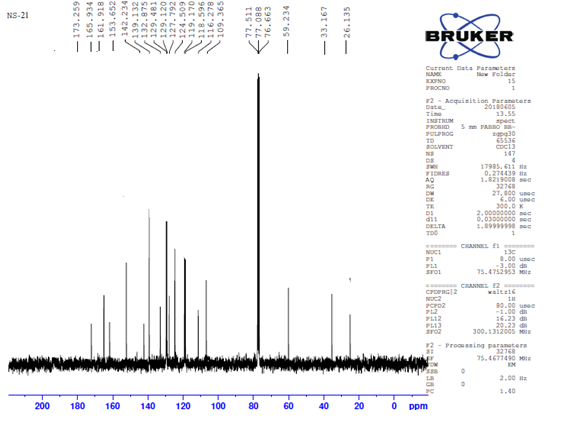


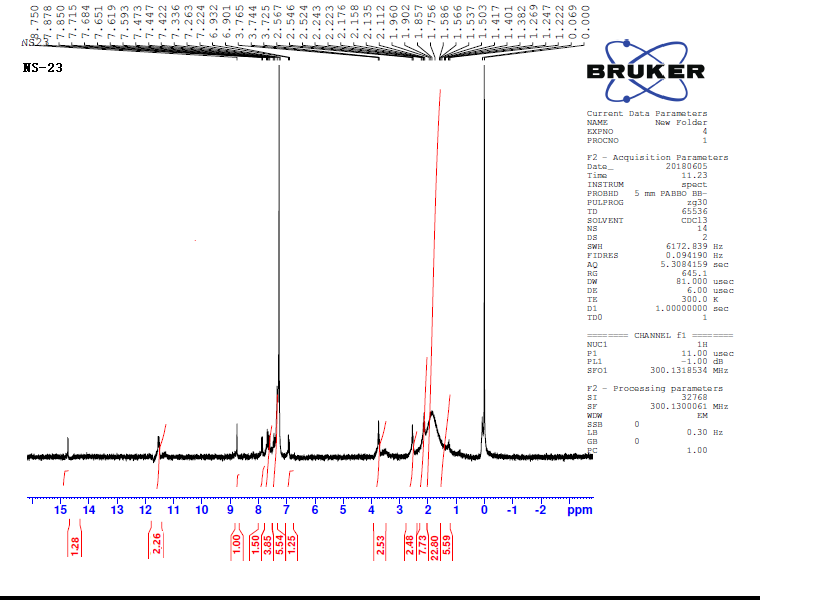

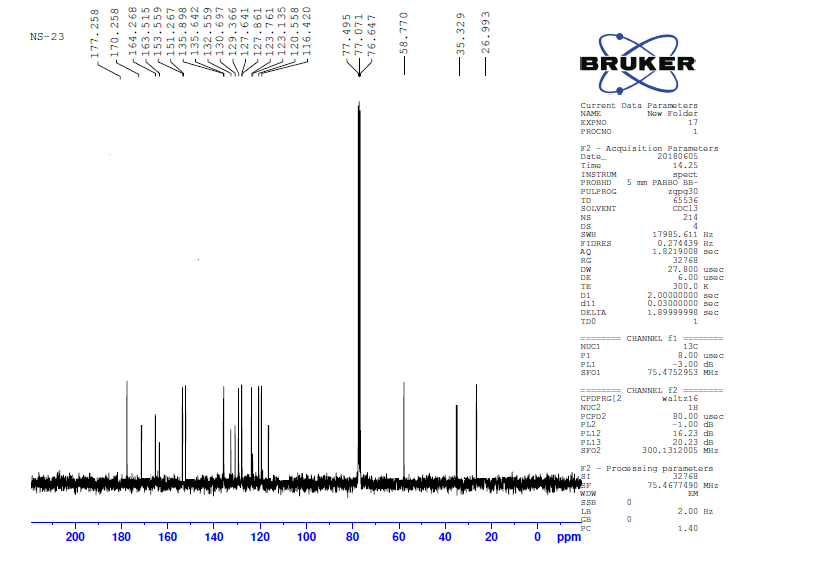

Supplement: Supplementary file 1 — Additional file 1. 1H and 13C NMR data of most active compounds has been provided. [file 13065_2019_558_MOESM1_ESM.docx]
